# The Dual Role of Glutamatergic Neurotransmission in Alzheimer’s Disease: From Pathophysiology to Pharmacotherapy

**DOI:** 10.3390/ijms21207452

**Published:** 2020-10-09

**Authors:** Vidyasagar Naik Bukke, Moola Archana, Rosanna Villani, Antonino Davide Romano, Agata Wawrzyniak, Krzysztof Balawender, Stanislaw Orkisz, Sarah Beggiato, Gaetano Serviddio, Tommaso Cassano

**Affiliations:** 1 Department of Clinical and Experimental Medicine, University of Foggia, 71122 Foggia, Italy; vidyasagar.bukke@unifg.it; 2 Department of Medical and Surgical Sciences, University of Foggia, 71122 Foggia, Italy; archana.moola@unifg.it (M.A.); rosanna.villani@unifg.it (R.V.); antonino.romano@unifg.it (A.D.R.); gaetano.serviddio@unifg.it (G.S.); 3 Morphological Science Department of Human Anatomy, Medical Faculty University of Rzeszów, 35-310 Rzeszów, Poland; a17041962@gmail.com (A.W.); balawender82@gmail.com (K.B.); sorkisz@ur.edu.pl (S.O.); 4 Department of Medical, Oral and Biotechnological Sciences, University of Chieti-Pescara, 66100 Chieti, Italy; bggsrh@unife.it

**Keywords:** glutamate, NMDA, AMPA, metabotropic receptors, EAAT1/2, therapeutic targets, glucose, ageing, amyoid-β, tau, AD

## Abstract

Alzheimer’s disease (AD) is an age-related dementia and neurodegenerative disorder, characterized by Aβ and tau protein deposition impairing learning, memory and suppressing synaptic plasticity of neurons. Increasing evidence suggests that there is a link between the glucose and glutamate alterations with age that down-regulates glucose utilization reducing glutamate levels in AD patients. Deviations in brain energy metabolism reinforce the development of AD by hampering glutamate levels in the brain. Glutamate is a nonessential amino acid and the major excitatory neurotransmitter synthesized from glucose. Alterations in cerebral glucose and glutamate levels precede the deposition of Aβ plaques. In the brain, over 40% of neuronal synapses are glutamatergic and disturbances in glutamatergic function have been implicated in pathophysiology of AD. Nevertheless, targeting the glutamatergic system seems to be a promising strategy to develop novel, improved therapeutics for AD. Here, we review data supporting the involvement of the glutamatergic system in AD pathophysiology as well as the efficacy of glutamatergic agents in this neurodegenerative disorder. We also discuss exciting new prospects for the development of improved therapeutics for this devastating disorder.

## 1. Introduction

Alzheimer’s disease (AD) is the most common and prevalent neurodegenerative disease with memory dysfunction and cognitive impairment, affecting nearly 46.8 million people worldwide, as reported by the World Health Organisation. AD is characterized by extracellular deposition of amyloid-β (Aβ) senile plaques and intracellular accumulation of neurofibrillary tangles (NFTs) [1]. Aβ is a short peptide that is produced from amyloid precursor protein (APP), a type I integral membrane glycoprotein which undergoes cleavage by both amyloidogenic and non-amyloidogenic pathways [2]. Amyloidogenic pathway produces soluble APPβ (sAPPβ) fragment, Aβ and APP intracellular domain (AICD) through sequential cleavage of APP by β-secretase and γ-secretase; non-amyloidogenic pathway generates soluble APPα (sAPPα), P3 peptide and AICD by α-secretase and γ-secretase [3]. Out of all Aβ species, soluble oligomeric Aβ1-42 is considered as the most neurotoxic product obtained after cleavage of APP [4]. Aβ was first identified in the extracellular component and later studies confirmed its presence in neuronal intracellular regions such as the endosome [5], endoplasmic reticulum [6], and trans-Golgi network [7]. Aβ in mitochondria reacts with proteins impairing oxidative phosphorylation and increasing reactive oxygen species (ROS) that damage the neuronal membrane [8,9,10].

Aβ peptides are involved in cognitive dysfunction by inducing synaptotoxicity through Aβ aggregation. Aβ species can be found at the synapse as low-molecular-weight (LMW) dimers, trimers and tetramers) and high-molecular-weight (HMW) oligomers [11]. HMW Aβ oligomers have greater binding affinity in hippocampal neuronal synapses when compared to LMW Aβ oligomers [12]. However, LMW Aβ oligomers induce more cognitive dysfunction and HMW Aβ oligomers cause a transient decrease in cognitive functions [13]. Nevertheless, HMW oligomers can dissociate into LMW species impairing synaptic functions [14] and in contrast, dissociation of Aβ oligomers into monomers in vivo could reduce Aβ pathology and synaptotoxicity [15]. Aβ oligomers target the excitatory synapses with greater affinity and alter the structure and functions of the synapses [16].

Tau is a microtubule-associated protein (MAP) engaged in the stability of microtubules in neurons and also regulates synaptic function [17]. Hyperphosphorylation of tau results in the formation of NFTs, which is a major pathological hallmark of AD. Tau phosphorylation acts as an indicator of aberrant activities of kinases and phosphatases in AD [18]. In dendrites, hyperphosphorylation of tau forms fibrils, which appear as neuropil threads and as NFTs in axon and somatodendritic section of neurons [19,20]. Tau protein undergoes phosphorylation by proline-directed protein kinases (PDPKs) or non-proline-directed protein kinases (non-PDPKs) [21]. PDPKs include Cyclin-Dependent Kinase-5 (Cdk-5), Glycogen synthase kinase-3 (GSK-3), and extracellular signal-related protein kinase (ERK). The irregular activity of Cdk-5 in AD causes tau hyperphosphorylation, loss of dendritic spines and deterioration of synaptic plasticity [22]. Non-PDPKs comprise of protein kinase A (PKA), Casein kinase 1 (CK1), and Casein kinase 2 (CK2) [18]. Among the kinases identified to be responsible for tau hyperphosphorylation, GSK-3β plays an important role in the pathological changes of tau protein in AD [23]. Data collected in humans show increased activation of GSK-3β in early-stage AD [24], while a consistent inhibition was observed in late-stage AD [25,26], thus suggesting that GSK-3β-mediated tau phosphorylation is among the earliest events during the progression of the pathology. Increasing evidence shows that tau in dendrites plays a key role in Aβ-induced detrimental effects. Of note, tau knockout mice showed a decrease in extrasynaptic *N*-methyl-d-aspartate (NMDA) receptor activity in the hippocampus contributing to neuroprotective effects [27]. Accumulation of Aβ plaques in synapse and the infiltration of tau into dendritic spines reduce excitatory glutamatergic synaptic transmission leading to cognitive impairments [28].

In the central nervous system (CNS), glutamate is the primary excitatory neurotransmitter acting on both ionotropic and metabotropic receptors. It is at the crossroad between multiple metabolic pathways and plays an important role in the functions of learning and memory. The activity of glutamatergic neurons is compromised in AD due to the destruction of synapse and neuronal death and its deficit can influence memory, cognition and behaviour, including cortical and hippocampal processing [29,30]. On the other hand, the pathological accumulation of glutamate can induce neurotoxicity due to time-related exposure, over-stimulating the post-synaptic response causing an increase in the entry of Ca^2+^ into neurons [31]. Since currently available therapies of AD include cholinesterase inhibitors, NMDA partial antagonist memantine, more information about AD-related changes in glutamate neurotransmission would be highly relevant to better understand its mechanisms of action and to optimize the treatment [32,33]. In fact, treatment using memantine has improved cognition, behaviour, global function but the degree of efficacy remains to be fully determined.

Thus, the present review will extensively cover recent findings on the dysregulation of glutamatergic signaling in AD and will highlight the molecular mechanisms through which the modulation of glutamatergic receptors might exert beneficial effects in AD treatment.

## 2. Glucose Levels Affect Glutamate Content in AD

AD patients commonly suffer from other co-morbidities (stress, depression, diabetes, renal disease, etc.) that increase the complexity of AD pathogenesis [34,35,36,37,38,39,40]. Among all the co-morbidities, diabetes remains the most prevailing and significant risk in developing AD [41,42,43,44,45,46]. Familial AD patients exhibit alterations in the cerebral glucose metabolism before the manifestation of amyloid plaques that might be the causative reason for AD development [47,48].

Glucose is the main energy substrate for the brain cells (glial cells and neurons). It is now well established that aberrations in cerebral glucose utilization, glycolysis and oxidative metabolism are associated with cognitive dysfunction in AD [43,48,49,50,51]. There is a particular pattern of regional brain glucose hypometabolism in AD affecting the parietal and temporal cortices, where the glucose deficit is on the order of 20–25% compared to age-matched, cognitively normal controls [52]. Many studies indicate that presymptomatic brain glucose hypometabolism can be present long before the threshold of cognitive symptoms of AD and could therefore potentially be contributing to the development and/or progression of both the cognitive decline and the neuropathological hallmarks associated with AD. To this regard, although a neuropathological link has not yet been fully demonstrated in humans, increasing evidence in transgenic mouse models of AD suggests that various experimental approaches to diminishing brain glucose supply all drive Aβ overproduction [53]. Moreover, both type-2 diabetes and its associated brain insulin resistance were proposed to favour tau hyper-phosphorylation in AD, although the molecular mechanisms are still unclear [51,54,55].

**Figure 1:**
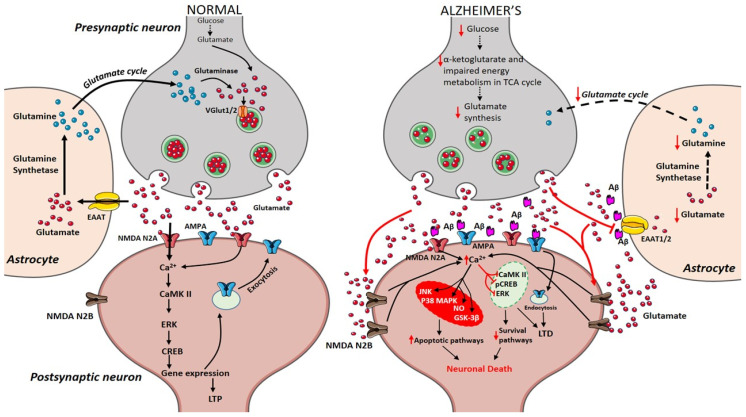
Presynaptic terminals release glutamate activating ionotropic receptors on postsynaptic neurons. In normal condition, *N*-methyl-d-aspartate (NMDA) NR2A activation induces increase in calcium levels favouring induction of long-term potentiation (LTP) through metabolic pathways (extracellular signal-related protein kinase (ERK), CaMK II, cyclic adenosine monophosphate response element-binding protein (CREB)). Excess glutamate left is taken up by astrocytes through EAAT2 converting into glutamine and glutamate by glutamine synthetase and glutaminase respectively (black arrows). The synthesized glutamate is transported into vesicles by VGlut1/2. Conversely in Alzheimer’s disease (AD), Aβ oligomers interfere with NMDA receptors increasing the spillover of glutamate (red arrows) to extrasynaptic sites activating NMDA NR2B receptors increasing excess calcium levels inhibiting prosurvival pathways. Imbalance in glutamate/glutamine cycle (black-dashed arrows) is also reported in the figure.

During the course of time, glucose alterations in the brain have a detrimental effect on the glutamatergic system due to an imbalance in glutamate availability. Neurons and glial cells work in tight cooperation in the glutamate/glutamine cycle and this cycle is connected with the energy metabolism, and, in turn, with the availability of glucose to the brain. Glutamate can be converted into α-ketoglutarate and vice-versa and serves as a substrate in the tricarboxylic acid (TCA) cycle. Research on APPswe/PSEN1dE9 mice showed that prior to amyloid plaque deposition, brain energy metabolism is affected leading to changes in glucose metabolism, reduced TCA cycle activity, decreased adenosine triphosphate (ATP) synthesis in isolated mitochondria were observed demonstrating that alterations in cerebral energy metabolism precede the amyloid plaques formation [56]. The conversion of glutamate to glutamine by the enzyme glutamine synthetase requires ATP and it has been estimated that 70% of the signaling energy comes from the oxidation of glucose [57]. Therefore, impairment in glucose metabolism impacts the glutamate receptor-mediated signal pathway leading to initial stages of memory impairment observed in patients affected by AD [58,59]. Figure 1 describes how alterations in cerebral glucose levels affect glutamate output leading to neuronal death in AD.

## 3. Glutamatergic System

### 3.1. Glutamate/Glutamine Cycle

Glutamate is a nonessential amino acid that does not cross the blood–brain barrier and is extensively distributed throughout the CNS. Glutamate is produced in neurons, and glial cells from the precursor’s glucose, α-ketoglutarate [60]. The concentration of glutamate in the synaptic cleft at resting condition is about 0.6 µM [61] and it goes to above 10 µM during presynaptic neuronal depolarisation, where overstimulation is prevented by the rapid and efficient removal from the extracellular space [62]. Glutamate is released into synapse from presynaptic neurons, leading to its uptake and activation of glutamatergic receptors. After receptor activation, the excess amount of glutamate is taken up by astrocytes through their excitatory amino acid transporters (EAAT) 1, and 2. In glial cells, glutamate is converted into glutamine in the presence of glutamine synthetase. Glutamine is a non-neuroactive molecule, which is released into extracellular space and lacks the ability to react with glutamate receptors [63]. Presynaptic neurons recover the glutamine and convert it into glutamate by the action of phosphate-activated glutaminase (PAG) [64]. Glutamate is transported into synaptic vesicles through the activity of vesicular glutamate transporters 1 and 2 (VGLUT1/2). This complete glutamate/glutamine cycle was established in the 1970’s and is described in Figure 1 [65,66].

Perturbations of the glutamate/glutamine cycle could cause excitotoxicity through the tonic activation of glutamate receptors, leading to an influx of Ca^2+^ that, in turn, can cause cell death by necrosis and apoptosis. In particular, the perturbation may be due to the inactivation of EAAT1/2 or glutamine synthetase, which would increase the concentration of glutamate in the synaptic cleft, causing over-excitation [67,68].

### 3.2. EAATs

EAATs take up the synaptic glutamate at synapse and terminate the glutamatergic transmission. Five mammalian EAAT isoforms (EAAT1–5) have been identified and EAAT1 and 2 are expressed mainly in astrocytes, while EAAT3, 4, and 5 are expressed in neurons [69,70,71]. However, EAAT2 is most significant in glutamate homeostasis and its dysfunction leads to glutamate-mediated toxicity and neuropathology in AD [67,68]. Electrophysiological studies confirm that 90–95% of extracellular glutamate is taken up by EAAT2 in astrocytes that modulate glutamate transmission and supply glutamate to other adjacent neurons [72]. Blockade with dihydrokainic acid (DHA), a selective inhibitor of EAAT2 [73] resulted in extended excitatory NMDA receptor-mediated synaptic current [74]. In astrocytes, glutamate undergoes oxidative metabolism producing ATP indicating that the glutamate transported via EAAT2 produces energy [75]. EAAT2 plays a vital role in cognitive functions [76], and its contribution to disease pathology is observed through the loss of EAAT2 protein and functions in AD patients [67]. Intriguingly, EAAT2 mRNA levels are not decreased in AD patients but the decline of EAAT2 protein levels indicates disturbances in the post-transcriptional process [68].

Moreover, mutation of APP/presenilin-1 in mice leads to a partial loss of EAAT2 expression, suggesting the imperative role of glia in AD pathology [77,78]. Moreover, the induction of Aβ1-42 leads to mislocalization of EAAT2 in glia, reducing the clearance of glutamate in the synaptic cleft [79]. EAAT2 undergoes impaired oxidation in AD patients and Aβ-treated rat cortical synaptosomes cultures [80]. In AD pathogenesis, lipid peroxidation in the neuronal membrane after Aβ deposition releases 4-Hydroxy-2-noneal (HNE), a neurotoxic peptide that promotes the generation of ROS modifying the structure and functions of EAAT2 [80,81]. HNE is an electrophilic aldehyde that reacts covalently with cysteine, lysine, and histidine residues of EAAT2 impairing its functions. This leads to an increase in glutamate concentration at the synaptic cleft triggering excitotoxicity mediated neurodegeneration [80,82]. Nonetheless, loss of EAAT2 function increases the activity of insulin-degrading enzymes in the liver, suggesting that the loss of EAATs causes insulin/protein kinase B signaling abnormalities in AD [83]. These findings suggest that EAAT2 loss/dysfunction associated with AD pathology and EAAT2 could be used as a therapeutic target for neuroprotection in glutamate-mediated excitotoxicity.

### 3.3. Ionotropic Receptors

Ionotropic glutamate receptors are ligand-gated ion channels that mediate fast excitatory transmission. They include three kinds of subfamilies named for their original selective agonists: NMDA, α-amino-3-hydroxy-5-methyl-4isoxasolepropionic acid (AMPA) and kainate [84]. Table 1 lists the receptor family members, subunits, signal pathway and relevant physiological function.

#### 3.3.1. NMDA Receptors

NMDA receptors are heterotetramers identified with seven different subunits such as one GluN1 subunit, four GluN2 subunits (GluN2A, GluN2B, GluN2C and GluN2D), and two GluN3 subunits (GluN3A and GluN3B) [103]. NMDA receptors comprise of two GluN1, two GluN2 or GluN3 subunits in some cases [104] and GluN1, is considered as an obligatory subunit. In addition to their synaptic localization, NMDA receptors are also found at extrasynaptic sites. In particular, extrasynaptic NMDA receptors are located on dendrites or on non-perisynaptic parts of the spine and require high glutamate concentrations in order to be activated [105]. These NMDA receptors are characterized by favoring the GluN2B subunit which, when excessively stimulated, contributes to pro-death signalling [106]. Moreover, extrasynaptic NMDA receptors are involved in the regulation of Aβ production and thus in the neuropathology of AD [107].

The activation of NMDA receptors is controlled by glutamate, D-serine or glycine binding and release of Mg^2+^ block after membrane depolarization [108]. NMDA receptors are permeable for Na^+^, K^+^, and highly permeable for Ca^2+^ that acts as a secondary messenger to stimulate intracellular signaling cascades such as Ca^2+^/calmodulin-dependent kinase II (CaMKII), ERK activation, and phosphorylation of cyclic adenosine monophosphate response element-binding protein (CREB), which are involved in inducing long-term potentiation (LTP) [109]. LTP induction promotes the growth of dendritic spines and the recruitment of AMPA receptors [110].

Though NMDA receptors maintain synaptic plasticity and survival of neurons, excess activation of NMDA receptors leads to excitotoxicity, neurodegeneration and cell death. The increase in Ca^2+^ levels activates catabolizing enzymes like calpain I [111], phospholipases [112] and arachidonic acid metabolism [113] resulting in a surge of reactive oxygen and nitrogen species leading to neuronal cytoskeleton collapse and membrane degeneration [114]. Moreover, elevated Ca^2+^ may also activate protein kinases, which lead to hyperphosphorylation of ubiquitin and tau [115,116]. Thus, modulation of NMDA receptor functions is beneficial in AD by regulating glutamate availability and altering NMDA receptor signaling [117].

#### 3.3.2. Interaction between NMDA Receptors and Aβ

**Figure 2:**
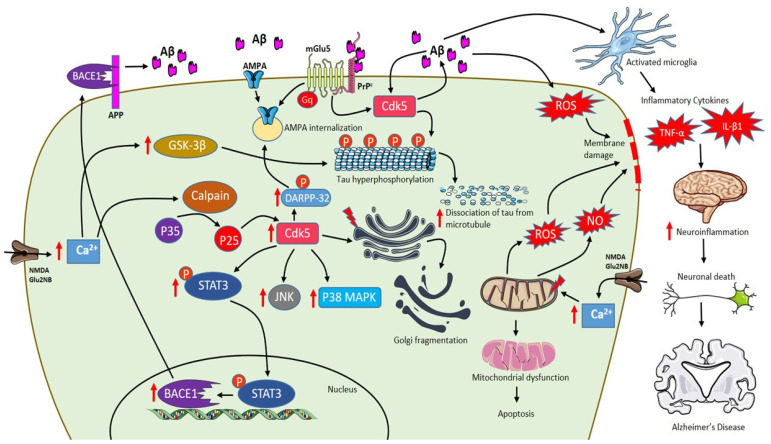
Schematic representation of AD pathophysiology involving Aβ, extrasynaptic NMDA Glu2NB, mGlu5 and role of activated microglia. Activation of NMDA Glu2NB receptors increases calcium levels inducing p35 to p25 cleavage mediated by calpain and p25 is activates cdk-5 enhancing DARPP-32 phosphorylation eventually leading to AMPA receptor internalization. Increase in Aβ levels activates Cdk-5 that plays a major role in Golgi fragmentation and tau phosphorylation leading to dissociation of tau from microtubules. Furthermore, Cdk-5 mediates phosphorylation of signal transducer and activator of transcription 3 (STAT3), which increases β-site APP cleaving enzyme 1 (BACE1) transcription resulting in an increase of Aβ content. Moreover, Aβ-PrPc-mGlu5 combination leads to AMPA internalization. Excess increase in levels of Ca^2+^ in mitochondria results in the generation of reactive oxygen species (ROS) and NO, inhibition of ATP synthesis, mitochondrial permeability transition pore (mPTP) opening, release of cytochrome c, activation of caspases and leading to apoptosis. In addition, Aβ also initiates a spectrum of neuroinflammation by activating microglia that plays a detrimental role in the expression of pro-inflammatory cytokines like interleukins and tumor necrosis factor-α (TNF-α) influencing neurodegeneration. (red arrows indicate “increase”).

Aβ peptides exist as monomers, dimers, trimers, tetramers, dodecamers, oligomers and protofibrils [118]. Studies confirm that soluble Aβ and oligomeric Aβ exert more toxic effects than insoluble amyloid plaques [119,120]. In the hippocampus and cortex, the brain region mainly affected by Aβ pathology in human, GluN2A and GluN2B are the predominant subunits with vital functions in synaptic plasticity [121]. Elevated Aβ peptides inhibit LTP leading to the shift of NMDA receptor-dependent signaling cascades to long-term depression (LTD) [110]. Therefore, the accumulation of Aβ oligomers disrupts the synaptic transmission leading to early cognitive deficits [122]. Aβ-induced LTD is caused by blocking glutamate uptake at glial cells, increasing glutamate levels in the synaptic cleft. Besides, Aβ peptides also enhance glutamate release from presynaptic neurons [123] and astrocytes [124]. Thus, an increase in glutamate level activates extra-synaptic GluN2B containing NMDA receptors, which cause a modest increase in postsynaptic Ca^2+^ triggering LTD and, in turn, leading to synaptic impairment due to the collapse of dendritic spines damaging synapse growth and plasticity [110,125]. In particular, a high level of Ca^2+^ activates p38 mitogen-activated protein kinases (MAPKs), GSK3β and c-Jun N-terminal kinase (JNK) pathways that are involved in cell death signaling and tau hyperphosphorylation [105,126,127]. The increase in cytosolic Ca^2+^ is rapidly taken up by mitochondria to prevent cytosolic Ca^2+^ overload. The excess levels of Ca^2+^ in mitochondria result in the generation of ROS and nitric oxide, inhibition of ATP synthesis, mitochondrial permeability transition pore (mPTP) opening, release of cytochrome c, activation of caspases and lead to apoptosis [128,129,130]. Moreover, Aβ also initiates a spectrum of neuroinflammation by activating microglia that plays a detrimental role in the expression of pro-inflammatory cytokines like interleukins and tumor necrosis factor-α (TNF-α) influencing neurodegeneration as shown in Figure 2 [131,132,133].

Several studies have demonstrated that Aβ is also able to modulate the expression of NMDA receptors and vice versa. In this regard, an in vivo study shows that a low-level activation of the NMDA receptor increases Aβ production, while the higher activation of the NMDA receptor decreases Aβ production [134]. Aβ oligomers also seem to bind the surface tyrosine kinase receptor namely the Ephrin ligand-receptor (EphB2), which maintains the integrity of NMDA receptors. Loss of NMDA receptor functions and reduction in LTP are noticed after EphB2 degradation that resulted in a decrease in NMDA receptor surface localization [135]. AD mouse models and post-mortem analysis of the prefrontal cortex in AD patients displayed an increase in expression of striatal-enriched protein tyrosine phosphatase 61 (STEP61). Aβ interacts with NMDA receptor reducing their surface expression through Aβ-induced NMDA receptor internalization by STEP61 through dephosphorylation of the GluN2B subunit at Tyr-1472 [136,137,138]. Aβ oligomers also react with cellular prion protein (PrPc) altering the NMDA receptor function through two known potential mechanisms [139]. Primarily, Aβ oligomer interacts with PrPc receptor activating Fyn, a tyrosine kinase phosphorylating GluN2B enhancing NMDA receptor function before dephosphorylation of GluN2B by increased levels of STEP61. Secondarily, Aβ chelates copper ions abrogating the binding of PrPc to NMDA receptors and inhibiting their activity thereby, producing large nondesensitizing steady-state NMDA receptor currents and neurotoxicity [140].

#### 3.3.3. Interaction between NMDA Receptors and Tau

Tau is an axonal protein engaged in the stability of microtubules and is a major component in NFT. Tau protein mis-localizes from axon to dendritic spines after phosphorylation resulting in synaptic impairment and LTP deficits [141]. NMDA receptor stimulation and Aβ deposition induce tau phosphorylation. Enhanced overexpression of tau phosphorylation may increase NMDA receptor transmission that could facilitate to LTD. NMDA receptor-dependent tau phosphorylation is reversible but Aβ-induced tau phosphorylation is not reversible if the Aβ exposure persists for more than five days [142]. Tau protein is involved in the activation of the NMDA receptor mediated by Fyn, a tyrosine kinase that phosphorylates GluN2B subunit at Tyr1472 stabilizing interaction with postsynaptic density protein 95 (PSD95) in dendritic spines [143]. Excess Fyn accompanies the excess tau in AD dendrites and upregulates NMDA receptor activity, flooding the dendrites with harmful levels of calcium. GluN2B and PSD95 complex stabilization increase glutamatergic excitotoxicity induced by Aβ [17,31,144]. Tau phosphorylation is reduced in the GluN2A subunit NMDA receptor through the protein kinase C (PKC)/GSK3 pathway [145]. Tau knockout disrupts enrichment of Fyn at postsynaptic density and phosphorylation of the GluN2B subunit of NMDA receptor that reduces toxicity induced by Aβ [146].

#### 3.3.4. AMPA Receptors

AMPA receptors mediate fast and excitatory synaptic neurotransmission in the brain. AMPA receptors are composed of tetrameric assemblies of two dimers having subunits GluA1-GluA4 [147]. AMPA receptors are expressed both in neural and glial cells, their tight control at synapse maintain synaptic plasticity. Aberrant trafficking of AMPA receptors impairs learning and memory [148].

#### 3.3.5. Interaction between AMPA Receptors and Aβ

AMPA receptors trafficking at the synapse is an important mechanism in inducing synaptic plasticity. Aberrant movement of AMPA receptors towards and away from synapse leads to impairment in learning and memory [148]. AD pathogenesis involves deviations in AMPA receptor trafficking pathways. Aβ plays an important role in promoting AMPA receptor endocytosis leading to synaptic depression [125]. Aβ induces p35 to p25 cleavage mediated by calpain in the hippocampus. P25 is a Cdk-5 activating peptide involved in the pathological action of Aβ [149]. P25/Cdk-5 activity is increased by Aβ that enhances phosphorylation at Thr-75 of dopamine-and cyclic adenosine monophosphate-regulated neuronal phosphoprotein (DARPP-32), which inhibits PKA activity [150]. Moreover, Aβ dephosphorylates Thr-34 of DARPP-32, which eventually leads to AMPA receptor internalization due to loss of GluA1 phosphorylation at Ser-845 [151]. Consistent with these findings, inhibition of p25 generation in 5xFAD transgenic mice rescues LTP and memory deficits [149]. In addition, activation of Cdk-5 demonstrated to increase Aβ production [152] and further studies revealed that Cdk-5 mediated phosphorylation of signal transducer and activator of transcription 3 (STAT3), which increases the transcription of β-site APP cleaving enzyme 1 (BACE1) [153], consequently resulting in an increase of Aβ generation as shown in Figure 2 [154]. Elevated Aβ levels stimulate Cdk-5 activity, which plays a major role in Golgi fragmentation [155] and tau phosphorylation leading to dissociation of tau from microtubules in AD [156].

Aβ prevents activation of Ca^+2^/CaMKII and their accumulation causes deviations in the redistribution of CaMKII from synapse to cytosol, preventing AMPA receptors insertion into the plasma membrane [151,157]. CaMKII has multiple roles in mediating AMPA receptor transmission and potentiating synaptic transmission through GluA1 phosphorylation at Ser-831 that increases AMPA receptor channel conductance, to facilitate AMPA receptor synaptic recruitment by TCR Gamma Alternate Reading Frame Protein (TARP), stargazin phosphorylation and potentiating Ras-ERK pathway [158,159,160]. APPswe transgenic mice and cultured neurons with Aβ exposure displayed consistent results confirming that AMPA receptor response decreases due to the redistribution of CaMKII [161].

### 3.4. Metabotropic Receptors

mGlu receptors are G protein-coupled receptors (GPCRs) classified into three subtypes containing eight mGlu receptors and are responsible for modulating and fine-tuning the synapse [162]. Group I mGlu receptors contain mGlu1 and mGlu2, which are linked to polyphosphoinositide (PI) hydrolysis and negatively coupled with K^+^ channels [163]. Group II mGlu receptors consist of mGlu3, mGlu5 and Group III comprise of mGlu4, mGlu6, mGlu7 and mGlu8. Group II and III negatively regulate adenylate cyclase but activate MAPKs and PI-3-kinase pathways [164,165].

All mGlu receptor subtypes are present in neurons, while mGlu3 and mGlu5 receptors are found in astrocytes and microglial cells express mGlu2, mGlu3 and mGlu5 receptors [166]. Table 1 lists the receptor family members, subunits, signal pathway and relevant physiological function.

#### 3.4.1. mGlu5 Receptors

mGlu5 receptors have been implicated in neurodegenerative diseases like Alzheimer’s, Parkinson’s and Huntington’s disease [167]. Memantine, a non-competitive NMDA receptor antagonist failed to attenuate Aβ-induced glutamate levels suggesting the involvement of other receptors [168]. Recent studies have demonstrated that mGlu5 but not mGlu1 mediates synaptotoxic signaling [169]. mGlu5 receptors are highly expressed in the brain cortex and hippocampal regions that play a preponderance role in cognition and hence assumed that mGlu5 receptors are involved in synaptotoxic effects of AD pathogenesis mediated by oligomeric forms of Aβ1-42 [170,171,172]. Deleterious effects of Aβ oligomers on mGlu5 receptors include receptor overactivation, intracellular Ca^2+^ accumulation, receptor clustering, impaired homeostasis and synaptic disruption [173,174].

mGlu5 receptors are linked to the extra-synaptic GluN2 subunit of the NMDA receptor and involved in regulating neuronal excitability possibly through Homer/PSD95/Shank protein complex [172,175,176]. mGlu5 receptors display peri-synaptic localization at the post-synaptic membrane [177], regulating neuronal excitability of ionotropic glutamate receptor channels [178,179]. Genetic deletion, antagonists and pharmacological blockade of mGlu5 receptors suppress excitotoxic degeneration and are found to be neuroprotective [180,181,182,183]. Moreover, mGlu5 receptor gene transfer into hippocampal cornu ammonis 1 (CA1) resulted in neurodegeneration, and in contrast, silencing mGlu5 receptors has shown protective effects in CA3 region of transgenic mice [184].

A study demonstrated that Aβ could suppress LTP in normal mice but not in mice lacking PrPc [139], suggesting that mGlu5 could be a co-receptor for both PrPc and Aβ oligomers [185]. mGlu5 receptor interacts with amino acids 91-153 of PrPc and is influenced by mGlu5 receptor ligands. Aβ-PrPc-mGlu5 combination signals Fyn tyrosine kinase activation and E2F phosphorylation leading to synaptic loss [186,187]. Furthermore, this combination causes the redistribution of CaMKII leading to AMPA internalization [151]. Besides, other studies also indicate that PrPc is essential for the actions of Aβ in altering mGlu5 functions to regulate synaptic plasticity [188]. PrPc is found to be a potential receptor for Aβ oligomers greater than 150 kDa [189]. Aβ oligomers increase the interaction of the mGlu5 receptor with PrPc mediating Aβ-dependent synaptotoxicity [186]. In AD mice, the deletion of mGlu5 receptors reduces amyloid content and rescues from memory deficits [181]. However, it was also shown that PrPc is not required for Aβ-induced synaptic depression, spine density reduction and blockade of LTP [190]. Consequently, it is necessary to evaluate the interactions of Aβ-PrPc-mGlu5 and their involvement in inducing synaptic depression.

#### 3.4.2. mGlu2/3 Receptors

mGlu2 receptors are located in presynaptic terminals of glutamatergic neurons inhibiting the release of glutamate and maintain glutamatergic transmission [94]. Meanwhile, mGlu3 receptors are present in the postsynaptic membrane and in glial cells [94,191]. mGlu3 receptor activation enhances neuroprotection by preventing glucose-induced oxidative injury through radical scavenging and antioxidant defence [192]. Selective activation of the mGlu2 receptor enhances neuronal vulnerability to Aβ, while dual activation of mGlu2 and mGlu3 receptors is protective against Aβ-induced neurotoxicity [193]. Therefore, a combination of mGlu2 receptor blockade and activation of the mGlu3 receptor could be used as a strategy in AD treatment [171]. mGlu receptors activation in microglia showed the controversial results that mGlu2 receptor activation intensifies myelin-induced neurotoxicity in cerebellar granule neurons, while neuroprotective effects are exerted by mGlu3 receptor agonist *N*-acetylaspartylglutamate (NAAG) [194]. Moreover, in astrocytes, mGlu3 receptor activation promotes non-amyloidogenic or α-cleavage of APP increasing sAPPα and inhibiting expression of β-secretase [195]. mGlu2 receptor activation induces microglial apoptosis [196], while another study shows that mGlu2 receptor mediates in Aβ clearance by microglia [197].

mGlu2 receptors are also involved in learning and cognition and its inhibition improved memory in AD rodent models [198,199]. mGlu2 receptor activation induces TNF-α mediating TNF receptor 1 and caspase-3 activation leading to microglial neurotoxicity [196]. Furthermore, upregulated levels of chromogranin-A peptide in AD after activation of group II metabotropic receptors results in microglial reactivity and neurotoxicity [200]. Of note, selective activation of mGlu2 receptor activation increases Aβ neurotoxicity while combined activation of mGlu2 and mGlu3 receptors found to be neuroprotective in the presence of astrocytes due to the release of Transforming growth factor β1 (TGF-β1) mediated by glial mGlu3 receptors [193]. TGF-β1 seems to play an imperative role in deploying synaptic plasticity and memory transition from early LTP to late LTP [201]. Moreover, TGF-β1 exerts neuroprotective and anti-inflammatory effects stimulating microglia in the clearance of Aβ peptides [202]. The connection between TGF-β1 gene polymorphism and AD strengthens the role of TGF-β1 in memory and neuroprotective functions [171]. Reduction in type-II TGF-β1 receptors and dysfunction in TGF-β1 signaling leads to neurodegeneration and AD pathology in mice [203,204].

mGlu3 receptor binds to peptide transmitter NAAG, the third most prevalent neurotransmitter after glutamate and gamma-aminobutyric acid (GABA) [205,206]. NAAG synthetase I (NAAGSI) mediates the synthesis of NAAG in neuronal cells. The glial enzyme, glutamate caboxypeptidase II (GCPII), converts NAAG into *N*-acetylaspartate (NAA) and glutamate, which gets transported into glial cells [206]. GCPII inhibitors increase the extracellular concentration of NAAG and the drugs that increase the NAAG levels are pro-cognitive in object recognition test [207]. Treatment with GCPII inhibitors, ZJ43 (*N*-[[[(1S)-1-Carboxy-3-methylbutyl]amino]carbonyl]-L-glutamic acid) and 2-(phosphonomethyl) pentanedioic acid (2-PMPA) improved cognition in mice. In aged triple transgenic mice, ZJ43 reversed the cognitive deficit and 2-PMPA improved short-term novel object recognition test in 9-month old AD mice [208]. NAAG is co-released along with glutamate into the synapse, while NAAG is released perisynaptically activating presynaptic mGlu3 receptors that inhibit further release of glutamate to prevent excitotoxicity and to reduce disease pathogenesis. Besides, mGlu3 receptor activation by NAAG also stimulates the release of TGF-β1 [206].

## 4. Therapeutics for AD

Drug discovery, research and development for AD are strenuous and challenging. Over hundreds of drugs have failed and, currently, there are 132 agents in clinical trials for AD treatment. Since 2003, no new drugs and no disease-modifying treatments (DMTs) have been approved for AD [209]. Current treatment of AD includes drugs targeting the cholinergic system such as donepezil, rivastigmine, galantamine; drug acting on the glutamatergic system like memantine and drugs that intervene both cholinergic and glutamatergic system namely Namzaric, a combination of memantine and donepezil [210]. Some of the therapeutic agents acting on the glutamatergic system are discussed below and listed in Table 2.

### 4.1. Modulators of Ionotropic Receptors

The non-competitive NMDA receptor antagonist memantine has an inhibitory effect on NMDA receptor-mediated excitotoxicity, improves cognition, slowdowns disease progression and reduces tau phosphorylation [32,33,211,226]. Phencyclidine, ketamine and MK-801 (dizocilpine) target NMDA receptors but their clinical applications are hampered due to severe side effects [214]. Memantine inhibits neuronal excitotoxicity by inhibiting extrasynaptic Ca^2+^ influx, improving symptoms in patients with moderate to severe AD [227]. However, memantine preferentially targets HMW Aβ-induced synaptotoxicity rescuing from both neuronal oxidative stress and transient memory impairment but unable to prevent LMW Aβ-induced persistent cognitive deficit [13]. An improved NMDA receptor antagonist nitromemantine protects neuronal synapses both in vitro and in vivo by selectively blocking the aberrant extrasynaptic activity over physiological synaptic NMDA receptor activity [124].

Uncaria rhynchophylla is a medicinal herb that contains oxindole alkaloid, rhynchophylline that restores LTP and alleviates Aβ-induced activation of extrasynaptic NMDA receptors. Rhynchophylline is a significant active compound that protects from deficits in spatial learning and memory induced by soluble Aβ oligomers. Moreover, rhynchophylline also prevents the hyperactivation of extrasynaptic NMDA receptors by reducing postsynaptic currents in AD mice [216]. Another medicinal plant Pulsatilla Chinensis contains a natural triterpenoid saponin compound anemoside A3 (AA3) that modulates synaptic connectivity and memory enhancement. AA3 increases serine phosphorylation of AMPA receptors subunit of GluA1 that is required for AMPA receptors trafficking at synapses. In the hippocampus, AA3 increases the expression of monoamine neurotransmitters and neurotrophin, a brain-derived neurotrophic factor. Furthermore, AA3 acts as a non-competitive NMDA receptor modulator protective against overexcitation and ischemic brain injury [217].

### 4.2. Modulators of mGlu Receptors

#### 4.2.1. mGlu5 Receptor Modulators

mGlu5 receptor is coupled with heterotrimeric G protein Gαq/11 and its activation results in the release of intracellular Ca^2+^ that is linked to numerous neurodegenerative diseases [167]. Genetic deletion of mGlu5 receptor rescues cognitive decline and AD pathogenesis in APPswe/PS1EΔ9 AD mouse model [181]. Besides, selective blockade of mGlu5 receptor activity with a negative allosteric modulator (NAM) 2-chloro-4-[2-[2,5-dimethyl-1-[4-(trifluoromethoxy)phe-nyl]imidazol-4-yl]ethynyl] pyridine (CTEP) improved cognition in AD mice [218]. CTEP rescues cognitive functions by decreasing Aβ levels through ZBTB16-mediated autophagy activation in APPswe/PS1EΔ9 mice [228]. However, the efficacy of CTEP remains inconsistent with disease progression. In 15-month-old APPswe/PS1EΔ9 mice, loss of CTEP efficacy is found after 36 weeks of treatment due to the abolished contribution of ZBTB16 and mammalian target of rapamycin (mTOR)-mediated signaling. This data suggests that the pathological role of mGlu5 receptors may shift during the course of disease progression and proper therapeutic strategies should be amended for beneficial outcomes [229].

Furthermore, the selective blockade of mGlu5 receptors with 3-((2-methyl-1,3-thiazol-4-yl)ethynyl)pyridine (MTEP) reversed the learning and memory deficits in AD mice by rescuing synaptic dysfunction [181,186]. A key finding suggests that the silent allosteric modulator (SAM) BMS-984923 was able to rescue the established memory deficits in AD mice with normal mGlu5 receptor signaling in APPswe/PS1EΔ9 mouse model of AD. BMS-984923 is able to potentially inhibit mGlu5-PrPc interactions preventing Aβ-induced pathological signaling [219]. Moreover, mGlu5 receptors are also involved in regulating the release of inflammatory factors and ATP in non-neuronal cells such as microglia and astrocytes [230]. Non-selective group I/II mGlu receptor antagonist LY341495, reported to improve synaptic plasticity and blocking Aβ-enhanced long-term depression [224]. Moreover, pretreatment with mGlu1/5 receptor agonist, 3,5-dihydroxyphenylglycone (DHPG) also decreased Aβ-enhanced LTD [220]. mGlu5 receptor non-competitive antagonist SIB1757, prevented Aβ-induced reduction of NMDA receptors when neurons were pretreated with this molecule [173].

#### 4.2.2. mGlu2/3 Receptor Agonist/Antagonists

mGlu2/3 receptor antagonists demonstrated pro-cognitive effects in the Morris water maze test [199], novel recognition test [231] and social recognition test [232]. Orthosteric mGlu2/3 receptor agonist like LY379268 exerts mGlu3 receptor signaling, protecting neurons through the production of TGF-β1 and glial cell line-derived neurotrophic factors (GDNF) [193,221,222,223]. Durand et al. (2017) showed that apart from mGlu3 receptor agonists, protective effects through astrocyte-derived neutrophins, neuronal mGlu3 receptor activation also protects against Aβ-induced toxicity that disagrees with a previous report by Caraci et al., 2011. LY379268 injection upregulates brain-derived neurotrophic factor (BDNF) mRNA and protein levels in neurons of the cerebral cortex and hippocampus [233], group II mGlu receptor agonist (2S,2′R,3′R)-2-(2′,3′-dicarboxycyclopropyl)glycine (DCG-IV) increased BDNF mRNA only in microglial cells [234], while another study found an increase in BDNF mRNA levels after treating cultured astrocytes with LY379268 [197]. mGlu2 receptor positive allosteric modulator (PAM), *N*-4’-cyano-biphenyl-3-yl)-*N*-(3 pyridinylmethyl)-ethanesulfonamide hydrochloride (LY566332) has increased Aβ-induced neurodegeneration, while this effect is prevented by treatment with mGlu2/3 receptor agonist (2S,1′S,2′S)-2-(9-xanthylmethyl)-2-(2′-carboxycyclopropyl) glycine (LY341495) [193].

### 4.3. EAAT2 Activators

EAAT2 impairment is implicated in excess glutamate accumulation at the synaptic cleft leading to neurodegeneration in AD. The compounds that increase the activity of EAAT2 may have therapeutic benefits and neuroprotection. To investigate the cognitive benefit of restored EAAT2 in APP_Sw,Ind_ mice, a novel translational activator (LDN/OSU-0212320) is used as a pharmacological approach that restored EAAT2 protein significantly with improved cognitive functions and reduced Aβ plaques [69]. In a virtual screening approach study, two molecules GT949 and GT951 were identified as PAM of EAAT2 in cultured cells that enhanced glutamate uptake showing neuroprotective properties [225].

## 5. Conclusions

Neurodegeneration as in AD is a multifactorial disease, which is why a general pathological mechanism and appropriate treatment have not been found, but various etiological hypotheses have been proposed in order to understand a little about the etiology of AD. To this regard, glutamate seems to play major roles in part because of its abundance in brain tissue and in part because it is at the crossroad of multiple metabolic pathways. In fact, glutamate is the major mediator of excitatory signals in SNC and both too much glutamate and too little glutamate are harmful. When this delicate balance is disrupted, the perturbations of glutamate neurotransmission have severe consequences, leading to the onset of AD. Although it has a pivotal role in the etiology of AD, the glutamatergic system offers many pharmacological tools and therapeutical targets in order to slow down the disease.

In this regard, antagonists of the NMDA receptor damper the excitotoxicity induced by glutamate in AD. Memantine and its combination with donepezil are approved by Food and Drug Administration to treat moderate to severe AD. However, these current medications are not able to completely rescue the brain cells from the damage of AD progression. Thus, there is a lot of need to focus on and develop some disease-modifying drugs that could slow the progression of AD. Some medicinal herbs contain active components, like rhynchophylline, and AA3, which restore LTP by inhibiting the activation of extrasynaptic NMDA receptor and enhance cognition by acting on AMPA receptors respectively. Moreover, mGlu2/3 ligands could be used as antagonists for AD treatment and mGlu5 receptor modulators rescue from cognitive decline. Of note, PAMs of EAAT2 could block glutamate-mediated excitotoxicity by increasing the glutamate clearance. Thus, it is clear that the future to treat AD is through a multi-drug and multi-model approach using combinations of potential drugs for the treatment. The lack of successful drug developments in AD has provided the opportunity to develop agents that could modify AD progression. Aiming at the glutamatergic system is one such target that could be beneficial in the treatment of AD by reducing glutamate levels rescuing from glutamate-induced excitotoxicity.

## Figures and Tables

**Table 1:** Overview of glutamatergic receptors manly involved in the pathophysiology and pharmacotherapy of AD.

IONOTROPIC RECEPTORS					
	**Subunits**	**Ions**	**Localization**	**Characteristic Functions**	**References**
NMDA	GluN1 GluN2A GluN2B GluN2C GluN2D GluN3A GluN3B	Ca^2+^, Na^+^	Postsynaptic neuron; GluN2B is primarily extra-synaptic	GluN1 is obligatory glycine binding subunit; Mg^2+^ block is removed upon depolarization for ion influx	[33,85,86,87,88,89,90]
AMPA	GluA1 GluA2 GluA3 GluA4	Ca^2+^, Na^+^	Presynaptic and postsynaptic neuron	Activation of presynaptic AMPA receptors results in a modulation of PKA activity; AMPA receptors depolarization in postsynaptic compartment leads to influx of Ca^2+^, Na^+^ ions activating NMDA receptors	[91,92,93]
**METABOTROPIC RECEPTORS (mGluRs)**					
**Groups**	**Receptors**	**G protein**	**Localization**	**Characteristic Functions**	**References**
Group 1 (Excitatory)	mGlu1 mGlu5	Gq	Primarily postsynaptic	Involved in mediating LTP and LTD; Enhances excitability and synaptic plasticity	[90,94,95,96,97]
Group 2 (Inhibitory)	mGlu2 mGlu3	Gi	Primarily presynaptic Primarily postsynaptic	mGluR2/3 receptors main function is to inhibit the release of glutamate. These receptors are activated by non-vesicular, extra-synaptic glutamate that is released by astrocytes in exchange for cystine	[90,94,97,98,99,100]
Group 3 (Inhibitory)	mGlu4 mGlu6 mGlu7 mGlu8	Gi	Pre- and postsynaptic Primarily postsynaptic Pre- and postsynaptic Primarily presynaptic	Inhibit the release of presynaptic glutamate; Inhibit NMDA activity and prevent neurotoxicity	[90,94,97,101,102]

**Table 2:** List of compounds acting on glutamatergic receptors, transporters and their key findings.

Receptors	Drugs	Mechanisms	Key Findings	Preclinical and Clinical Studies/Approval Status	References
NMDA	Memantine	Non-competitive NMDAR antagonist	Improves cognition Slow-down disease progression Reduce tau phosphorylation	Approved by EMEA in 2002 and by USFDA in 2003	[32,33,211,212,213]
	Phencyclidine	NMDAR antagonist	Psychotomimetic	No clinical applications	[214]
	Ketamine	Non-competitive NMDAR antagonist	Psychotomimetic	No clinical applications	[214]
	MK-801 (Dizocilpine)	Non-competitive NMDA blocker	Cardiovascular side effects	No clinical applications	[214]
	Nitromemantine	Selective inhibition of extrasynaptic NMDAR	Ameliorates Aβ-induced synaptic loss	In vivo studies in α7nAChR-knockout, hAPP-J20 Tg, and 3× Tg AD mice	[124]
	Rhynchophylline (oxindole alkaloid)	Prevented excessive activation of Aβ_1-42_-induced postsynaptic extrasynaptic NMDARs	Rescues Aβ1–42-induced spatial dysfunction and LTP impairment. Limited application due to low water solubility, low concentration in brain tissue and low bioavailability	In vivo studies in Adult male Sprague-Dawley rats and C57BL/6 mice are under research to improve brain targeted delivery	[215,216]
AMPA	Anemoside A3 (triterpenoid saponin)	Modulates AMPA receptor	Improves memory and synaptic strength	In vivo studies in C57BL/6 (C57) mice	[217]
mGluR5	2-chloro-4-[2-[2,5-dimethyl-1-[4-(trifluoromethoxy)phe-nyl]imidazol-4-yl]ethynyl] pyridine (CTEP)	Negative Allosteric Modulator	Improves cognition	In vivo studies and CTEP is analogue of phase II molecule Basimglurant (RO4917523)	[218]
	3-((2-methyl-1,3-thiazol- 4-yl)ethynyl)pyridine (MTEP)	mGluR5-specific antagonist	Rescues from synaptic dysfunction and ameliorates learning and memory	In vivo studies in APPswe/PS1ΔE9 mice, APP/PS1 transgenic mice	[181,186]
	BMS-984923	Silent Allosteric modulator-	Rescues memory deficits and prevents Aβ-induced pathological signaling	In vitro studies in APPswe/PS1DE9 (APP/PS1) transgenic model mice In vivo studies in HEK293T cells	[219]
	LY341495	Non-selective group I/II mGluR antagonist	Completely blocks Aβ-induced LTD	In vivo studies in male Wistar rats	[220]
	3,5-dihydroxyphenylglycone (DHPG)	Group 1 mGluR (mGluR1/5) agonist	Prevents Aβ-induced LTD	In vivo studies in male Wistar rats	[220]
	SIB1757-[6-methyl-2-(phenylazo)-3-pyridinol]	Noncompetitive mGluR5 antagonist	Prevents Aβ-induced reduction of NMDARs	In vivo studies in mGluR5 knockout mice	[173]
mGluR2/3	LY379268	Orthosteric mGluR2/3 agonists	Protects neurons through TGF-β and GDNF production	In Mixed Cultures of Mouse Cortical Cells; mGlu2 and mGlu3 receptor knockout mice	[193,221,222,223]
	LY341495	mGluR2/3 antagonists	Blocks release of Aβ42	In cultured astrocytes and cultured neurons lacking mGlu2 receptors; TgCRND8 mice overexpressing a mutant human APP 695	[193,224]
EAAT2	LDN/OSU-0212320	EAAT2 translational activator	Improves cognitive functions	In vivo studies in APP_Sw,Ind_ mice	[69]
	GT949, GT951	Positive allosteric modulators	Enhances glutamate transport	In vivo studies in C57BL/6 mice	[225]

## Abbreviations

AA3	Anemoside A3
AD	Alzheimer’s disease
AICD	APP intracellular domain
AMPA	α-amino-3-hydroxy-5-methyl-4isoxasolepropionic acid
ATP	Adenosine triphosphate
APP	Amyloid precursor protein
Aβ	Amyloid-β
BDNF	Brain-derived neurotrophic factor
BACE1	β-site APP cleaving enzyme 1
CA1	cornu ammonis 1
CAMKII	Calmodulin-dependent kinase II
Cdk-5	Cyclin Dependent Kinase-5
CK1/2	Casein kinase 1/2
CNS	Central nervous system
CREB	Cyclic adenosine monophosphate response element binding protein
CTEP	2-chloro-4-[2-[2,5-dimethyl-1-[4-(trifluoromethoxy)phe-nyl]imidazol-4-yl]ethynyl] pyridine
DARPP-32	Dopamine and cyclic adenosine monophosphate-regulated neuronal phosphoprotein
DCG-IV	(2S,2′R,3′R)-2-(2′,3′-dicarboxycyclopropyl)glycine
DHA	Dihydrokainic acid
DHPG	3,5-dihydroxyphenylglycone
EAATs	Excitatory aminoacid transporters
EphB2	Ephrin ligand receptor
ERK	Extracellular signal-related kinase
GABA	Gamma-Aminobutyric acid
GCPII	Glutamate caboxypeptidase II
GDNF	Glial cell line-derived neurotrophic factors
GPCRs	G protein-coupled receptors
GSK3β	Glycogen synthase kinase 3-β
HMW	High-molecular-weight
HNE	4-Hydroxy-2-noneal
JNK	c-Jun N-terminal kinase
LTD	Long-term depression
LTP	Long-term potentiation
LMW	Low-molecular-weight
mTOR	mammalian Target of Rapamycin
MAP	Microtubule associated protein
MAPK	Mitogen-activated protein kinase
mPTP	mitochondrial permeability transition pore
MTEP	3-((2-methyl-1,3-thiazol-4-yl)ethynyl)pyridine
NAA	*N*-acetylaspartate
NAAG	*N*-acetylaspartylglutamate
NAAGS-I	NAAG synthetase I
NAM	Negative allosteric modulator
NFTs	Neurofibrillary tangles
NMDA	*N*-methyl-D-aspartate
Non-PDPKs	Non-proline-directed protein kinases
PAG	Phosphate-activated glutaminase
PAM	Positive allosteric modulator
PSD95	Postsynaptic density protein 95
PDPKs	Proline-directed protein kinases
PI	Polyphosphoinositide
PKA	Protein kinase A
PrPc	Cellular prion protein
ROS	Reactive oxygen species
SAM	Silent allosteric modulator
sAPPα	Soluble APPα
sAPPβ	Soluble APPβ
STAT3	Signal transducer and activator of transcription 3
STEP61	Striatal-enriched protein tyrosine phosphatase 61
TARP	TCR Gamma Alternate Reading Frame Protein
TCA	Tricaboxylic acid
TGF-β1	Transforming growth factor β1
TMD	Transmembrane domain
TNF-α	Tumor necrosis factor-α
VGLUT1/2	Vesicular glutamate transporters 1 and 2
2-PMPA	2-(phosphonomethyl) pentanedioic acid

## References

[1] Querfurth H.W., LaFerla F.M. (2010). Alzheimer’s disease. N. Engl. J. Med..

[2] Chen G.F., Xu T.H., Yan Y., Zhou Y.R., Jiang Y., Melcher K., Xu H.E. (2017). Amyloid beta: Structure, biology and structure-based therapeutic development. Acta Pharmacol. Sin..

[3] Coronel R., Palmer C., Bernabeu-Zornoza A., Monteagudo M., Rosca A., Zambrano A., Liste I. (2019). Physiological effects of amyloid precursor protein and its derivatives on neural stem cell biology and signaling pathways involved. Neural Regen. Res..

[4] Larson M.E., Lesne S.E. (2012). Soluble Abeta oligomer production and toxicity. J. Neurochem..

[5] Rogaeva E., Meng Y., Lee J.H., Gu Y., Kawarai T., Zou F., Katayama T., Baldwin C.T., Cheng R., Hasegawa H. (2007). The neuronal sortilin-related receptor SORL1 is genetically associated with Alzheimer disease. Nat. Genet..

[6] Cook D.G., Forman M.S., Sung J.C., Leight S., Kolson D.L., Iwatsubo T., Lee V.M., Doms R.W. (1997). Alzheimer’s A beta(1-42) is generated in the endoplasmic reticulum/intermediate compartment of NT2N cells. Nat. Med..

[7] Greenfield J.P., Tsai J., Gouras G.K., Hai B., Thinakaran G., Checler F., Sisodia S.S., Greengard P., Xu H. (1999). Endoplasmic reticulum and trans-Golgi network generate distinct populations of Alzheimer beta-amyloid peptides. Proc. Natl. Acad. Sci. USA.

[8] Cassano T., Pace L., Bedse G., Lavecchia A.M., De Marco F., Gaetani S., Serviddio G. (2016). Glutamate and Mitochondria: Two Prominent Players in the Oxidative Stress-Induced Neurodegeneration. Curr. Alzheimer Res..

[9] Pagani L., Eckert A. (2011). Amyloid-Beta interaction with mitochondria. Int. J. Alzheimer’s Dis..

[10] Serviddio G., Romano A.D., Cassano T., Bellanti F., Altomare E., Vendemiale G. (2011). Principles and therapeutic relevance for targeting mitochondria in aging and neurodegenerative diseases. Curr. Pharm. Des..

[11] Forner S., Baglietto-Vargas D., Martini A.C., Trujillo-Estrada L., LaFerla F.M. (2017). Synaptic Impairment in Alzheimer’s Disease: A Dysregulated Symphony. Trends Neurosci..

[12] Velasco P.T., Heffern M.C., Sebollela A., Popova I.A., Lacor P.N., Lee K.B., Sun X., Tiano B.N., Viola K.L., Eckermann A.L. (2012). Synapse-binding subpopulations of Abeta oligomers sensitive to peptide assembly blockers and scFv antibodies. ACS Chem. Neurosci..

[13] Figueiredo C.P., Clarke J.R., Ledo J.H., Ribeiro F.C., Costa C.V., Melo H.M., Mota-Sales A.P., Saraiva L.M., Klein W.L., Sebollela A. (2013). Memantine rescues transient cognitive impairment caused by high-molecular-weight abeta oligomers but not the persistent impairment induced by low-molecular-weight oligomers. J. Neurosci. Off. J. Soc. Neurosci..

[14] Yang T., Li S., Xu H., Walsh D.M., Selkoe D.J. (2017). Large Soluble Oligomers of Amyloid beta-Protein from Alzheimer Brain Are Far Less Neuroactive Than the Smaller Oligomers to Which They Dissociate. J. Neurosci. Off. J. Soc. Neurosci..

[15] Kim H.Y., Kim H.V., Jo S., Lee C.J., Choi S.Y., Kim D.J., Kim Y. (2015). EPPS rescues hippocampus-dependent cognitive deficits in APP/PS1 mice by disaggregation of amyloid-beta oligomers and plaques. Nat. Commun..

[16] Koffie R.M., Meyer-Luehmann M., Hashimoto T., Adams K.W., Mielke M.L., Garcia-Alloza M., Micheva K.D., Smith S.J., Kim M.L., Lee V.M. (2009). Oligomeric amyloid beta associates with postsynaptic densities and correlates with excitatory synapse loss near senile plaques. Proc. Natl. Acad. Sci. USA.

[17] Ittner L.M., Ke Y.D., Delerue F., Bi M., Gladbach A., van Eersel J., Wolfing H., Chieng B.C., Christie M.J., Napier I.A. (2010). Dendritic function of tau mediates amyloid-beta toxicity in Alzheimer’s disease mouse models. Cell.

[18] Hanger D.P., Anderton B.H., Noble W. (2009). Tau phosphorylation: The therapeutic challenge for neurodegenerative disease. Trends Mol. Med..

[19] Gotz J., Probst A., Spillantini M.G., Schafer T., Jakes R., Burki K., Goedert M. (1995). Somatodendritic localization and hyperphosphorylation of tau protein in transgenic mice expressing the longest human brain tau isoform. EMBO J..

[20] Uchihara T., Nakamura A., Yamazaki M., Mori O. (2001). Evolution from pretangle neurons to neurofibrillary tangles monitored by thiazin red combined with Gallyas method and double immunofluorescence. Acta Neuropathol..

[21] Wang Y., Mandelkow E. (2016). Tau in physiology and pathology. Nat. Rev. Neurosci..

[22] Kimura T., Ishiguro K., Hisanaga S. (2014). Physiological and pathological phosphorylation of tau by Cdk5. Front. Mol. Neurosci..

[23] Medina M., Garrido J.J., Wandosell F.G. (2011). Modulation of GSK-3 as a Therapeutic Strategy on Tau Pathologies. Front. Mol. Neurosci..

[24] Nicolia V., Ciraci V., Cavallaro R.A., Ferrer I., Scarpa S., Fuso A. (2017). GSK3β 5′-flanking DNA Methylation and Expression in Alzheimer’s Disease Patients. Curr. Alzheimer Res..

[25] Tramutola A., Triplett J.C., Di Domenico F., Niedowicz D.M., Murphy M.P., Coccia R., Perluigi M., Butterfield D.A. (2015). Alteration of mTOR signaling occurs early in the progression of Alzheimer disease (AD): Analysis of brain from subjects with pre-clinical AD, amnestic mild cognitive impairment and late-stage AD. J. Neurochem..

[26] Yarchoan M., Toledo J.B., Lee E.B., Arvanitakis Z., Kazi H., Han L.Y., Louneva N., Lee V.M., Kim S.F., Trojanowski J.Q. (2014). Abnormal serine phosphorylation of insulin receptor substrate 1 is associated with tau pathology in Alzheimer’s disease and tauopathies. Acta Neuropathol..

[27] Pallas-Bazarra N., Draffin J., Cuadros R., Antonio Esteban J., Avila J. (2019). Tau is required for the function of extrasynaptic NMDA receptors. Sci. Rep..

[28] Zhao X., Kotilinek L.A., Smith B., Hlynialuk C., Zahs K., Ramsden M., Cleary J., Ashe K.H. (2016). Caspase-2 cleavage of tau reversibly impairs memory. Nat. Med..

[29] Cassano T., Serviddio G., Gaetani S., Romano A., Dipasquale P., Cianci S., Bellanti F., Laconca L., Romano A.D., Padalino I. (2012). Glutamatergic alterations and mitochondrial impairment in a murine model of Alzheimer disease. Neurobiol. Aging.

[30] Rupsingh R., Borrie M., Smith M., Wells J.L., Bartha R. (2011). Reduced hippocampal glutamate in Alzheimer disease. Neurobiol. Aging.

[31] Liu J., Chang L., Song Y., Li H., Wu Y. (2019). The Role of NMDA Receptors in Alzheimer’s Disease. Front. Neurosci..

[32] Lipton S.A. (2006). Paradigm shift in neuroprotection by NMDA receptor blockade: Memantine and beyond. Nat. Rev. Drug Discov..

[33] Parsons C.G., Stoffler A., Danysz W. (2007). Memantine: A NMDA receptor antagonist that improves memory by restoration of homeostasis in the glutamatergic system—Too little activation is bad, too much is even worse. Neuropharmacology.

[34] Aubert L., Pichierri S., Hommet C., Camus V., Berrut G., de Decker L. (2015). Association between comorbidity burden and rapid cognitive decline in individuals with mild to moderate Alzheimer’s disease. J. Am. Geriatr. Soc..

[35] Doraiswamy P.M., Leon J., Cummings J.L., Marin D., Neumann P.J. (2002). Prevalence and impact of medical comorbidity in Alzheimer’s disease. J. Gerontol. A Biol. Sci. Med. Sci..

[36] Bellanti F., Iannelli G., Blonda M., Tamborra R., Villani R., Romano A., Calcagnini S., Mazzoccoli G., Vinciguerra M., Gaetani S. (2017). Alterations of Clock Gene RNA Expression in Brain Regions of a Triple Transgenic Model of Alzheimer’s Disease. J. Alzheimer’s Dis..

[37] Cassano T., Calcagnini S., Carbone A., Bukke V.N., Orkisz S., Villani R., Romano A., Avolio C., Gaetani S. (2019). Pharmacological Treatment of Depression in Alzheimer’s Disease: A Challenging Task. Front. Pharmacol..

[38] Cassano T., Magini A., Giovagnoli S., Polchi A., Calcagnini S., Pace L., Lavecchia M.A., Scuderi C., Bronzuoli M.R., Ruggeri L. (2019). Early intrathecal infusion of everolimus restores cognitive function and mood in a murine model of Alzheimer’s disease. Exp. Neurol..

[39] Cassano T., Romano A., Macheda T., Colangeli R., Cimmino C.S., Petrella A., LaFerla F.M., Cuomo V., Gaetani S. (2011). Olfactory memory is impaired in a triple transgenic model of Alzheimer disease. Behav. Brain Res..

[40] Romano A., Pace L., Tempesta B., Lavecchia A.M., Macheda T., Bedse G., Petrella A., Cifani C., Serviddio G., Vendemiale G. (2014). Depressive-like behavior is paired to monoaminergic alteration in a murine model of Alzheimer’s disease. Int. J. Neuropsychopharmacol..

[41] Arvanitakis Z., Wilson R.S., Bienias J.L., Evans D.A., Bennett D.A. (2004). Diabetes mellitus and risk of Alzheimer disease and decline in cognitive function. Arch. Neurol..

[42] Barone E., Tramutola A., Triani F., Calcagnini S., Di Domenico F., Ripoli C., Gaetani S., Grassi C., Butterfield D.A., Cassano T. (2019). Biliverdin Reductase-A Mediates the Beneficial Effects of Intranasal Insulin in Alzheimer Disease. Mol. Neurobiol..

[43] Bedse G., Di Domenico F., Serviddio G., Cassano T. (2015). Aberrant insulin signaling in Alzheimer’s disease: Current knowledge. Front. Neurosci..

[44] Fukazawa R., Hanyu H., Sato T., Shimizu S., Koyama S., Kanetaka H., Sakurai H., Iwamoto T. (2013). Subgroups of Alzheimer’s disease associated with diabetes mellitus based on brain imaging. Dement. Geriatr. Cogn. Disord..

[45] Ott A., Stolk R.P., van Harskamp F., Pols H.A., Hofman A., Breteler M.M. (1999). Diabetes mellitus and the risk of dementia: The Rotterdam Study. Neurology.

[46] Sharma N., Tramutola A., Lanzillotta C., Arena A., Blarzino C., Cassano T., Butterfield D.A., Di Domenico F., Perluigi M., Barone E. (2019). Loss of biliverdin reductase-A favors Tau hyper-phosphorylation in Alzheimer’s disease. Neurobiol. Dis..

[47] Gibson G.E., Shi Q. (2010). A mitocentric view of Alzheimer’s disease suggests multi-faceted treatments. J. Alzheimer’s Dis..

[48] Mosconi L., Pupi A., De Leon M.J. (2008). Brain glucose hypometabolism and oxidative stress in preclinical Alzheimer’s disease. Ann. N. Y. Acad. Sci..

[49] Barone E., Di Domenico F., Cassano T., Arena A., Tramutola A., Lavecchia M.A., Coccia R., Butterfield D.A., Perluigi M. (2016). Impairment of biliverdin reductase-A promotes brain insulin resistance in Alzheimer disease: A new paradigm. Free Radic. Biol. Med..

[50] Blass J.P., Sheu R.K., Gibson G.E. (2000). Inherent abnormalities in energy metabolism in Alzheimer disease. Interaction with cerebrovascular compromise. Ann. N. Y. Acad Sci..

[51] Pardeshi R., Bolshette N., Gadhave K., Ahire A., Ahmed S., Cassano T., Gupta V.B., Lahkar M. (2017). Insulin signaling: An opportunistic target to minify the risk of Alzheimer’s disease. Psychoneuroendocrinology.

[52] Cunnane S., Nugent S., Roy M., Courchesne-Loyer A., Croteau E., Tremblay S., Castellano A., Pifferi F., Bocti C., Paquet N. (2011). Brain fuel metabolism, aging, and Alzheimer’s disease. Nutrition.

[53] Velliquette R.A., O’Connor T., Vassar R. (2005). Energy inhibition elevates beta-secretase levels and activity and is potentially amyloidogenic in APP transgenic mice: Possible early events in Alzheimer’s disease pathogenesis. J. Neurosci..

[54] Van der Harg J.M., Eggels L., Bangel F.N., Ruigrok S.R., Zwart R., Hoozemans J.J.M., la Fleur S.E., Scheper W. (2017). Insulin deficiency results in reversible protein kinase A activation and tau phosphorylation. Neurobiol. Dis..

[55] Zhang X., Tang S., Zhang Q., Shao W., Han X., Wang Y., Du Y. (2016). Endoplasmic reticulum stress mediates JNK-dependent IRS-1 serine phosphorylation and results in Tau hyperphosphorylation in amyloid β oligomer-treated PC12 cells and primary neurons. Gene.

[56] Andersen J.V., Christensen S.K., Aldana B.I., Nissen J.D., Tanila H., Waagepetersen H.S. (2017). Alterations in Cerebral Cortical Glucose and Glutamine Metabolism Precedes Amyloid Plaques in the APPswe/PSEN1dE9 Mouse Model of Alzheimer’s Disease. Neurochem. Res..

[57] Dienel G.A. (2019). Brain Glucose Metabolism: Integration of Energetics with Function. Physiol. Rev..

[58] Hoyer S. (2004). Glucose metabolism and insulin receptor signal transduction in Alzheimer disease. Eur. J. Pharmacol..

[59] Iwangoff P., Armbruster R., Enz A., Meier-Ruge W. (1980). Glycolytic enzymes from human autoptic brain cortex: Normal aged and demented cases. Mech. Ageing Dev..

[60] Frisardi V., Panza F., Farooqui A.A. (2011). Late-life depression and Alzheimer’s disease: The glutamatergic system inside of this mirror relationship. Brain Res. Rev..

[61] Bouvier M., Szatkowski M., Amato A., Attwell D. (1992). The glial cell glutamate uptake carrier countertransports pH-changing anions. Nature.

[62] Clements J.D., Lester R.A., Tong G., Jahr C.E., Westbrook G.L. (1992). The time course of glutamate in the synaptic cleft. Science.

[63] Conway M.E. (2020). Alzheimer’s disease: Targeting the glutamatergic system. Biogerontology.

[64] Walton H.S., Dodd P.R. (2007). Glutamate-glutamine cycling in Alzheimer’s disease. Neurochem. Int..

[65] Hertz L. (1979). Functional interactions between neurons and astrocytes I. Turnover and metabolism of putative amino acid transmitters. Prog. Neurobiol..

[66] Van den Berg C.J., Garfinkel D. (1971). A simulation study of brain compartments. Metabolism of glutamate and related substances in mouse brain. Biochem. J..

[67] Jacob C.P., Koutsilieri E., Bartl J., Neuen-Jacob E., Arzberger T., Zander N., Ravid R., Roggendorf W., Riederer P., Grunblatt E. (2007). Alterations in expression of glutamatergic transporters and receptors in sporadic Alzheimer’s disease. J. Alzheimer’s Dis..

[68] Li S., Mallory M., Alford M., Tanaka S., Masliah E. (1997). Glutamate transporter alterations in Alzheimer disease are possibly associated with abnormal APP expression. J. Neuropathol. Exp. Neurol.

[69] Takahashi K., Foster J.B., Lin C.L. (2015). Glutamate transporter EAAT2: Regulation, function, and potential as a therapeutic target for neurological and psychiatric disease. Cell Mol. Life Sci..

[70] Castaldo P., Magi S., Cataldi M., Arcangeli S., Lariccia V., Nasti A.A., Ferraro L., Tomasini M.C., Antonelli T., Cassano T. (2010). Altered regulation of glutamate release and decreased functional activity and expression of GLT1 and GLAST glutamate transporters in the hippocampus of adolescent rats perinatally exposed to Delta(9)-THC. Pharmacol. Res..

[71] Castaldo P., Magi S., Gaetani S., Cassano T., Ferraro L., Antonelli T., Amoroso S., Cuomo V. (2007). Prenatal exposure to the cannabinoid receptor agonist WIN 55,212-2 increases glutamate uptake through overexpression of GLT1 and EAAC1 glutamate transporter subtypes in rat frontal cerebral cortex. Neuropharmacology.

[72] Fontana A.C. (2015). Current approaches to enhance glutamate transporter function and expression. J. Neurochem..

[73] Tse D.Y., Chung I., Wu S.M. (2014). Pharmacological inhibitions of glutamate transporters EAAT1 and EAAT2 compromise glutamate transport in photoreceptor to ON-bipolar cell synapses. Vision Res..

[74] Lozovaya N.A., Kopanitsa M.V., Boychuk Y.A., Krishtal O.A. (1999). Enhancement of glutamate release uncovers spillover-mediated transmission by N-methyl-D-aspartate receptors in the rat hippocampus. Neuroscience.

[75] McKenna M.C. (2012). Substrate competition studies demonstrate oxidative metabolism of glucose, glutamate, glutamine, lactate and 3-hydroxybutyrate in cortical astrocytes from rat brain. Neurochem. Res..

[76] Heo S., Jung G., Beuk T., Hoger H., Lubec G. (2012). Hippocampal glutamate transporter 1 (GLT-1) complex levels are paralleling memory training in the Multiple T-maze in C57BL/6J mice. Brain Struct. Funct..

[77] Mookherjee P., Green P.S., Watson G.S., Marques M.A., Tanaka K., Meeker K.D., Meabon J.S., Li N., Zhu P., Olson V.G. (2011). GLT-1 loss accelerates cognitive deficit onset in an Alzheimer’s disease animal model. J. Alzheimer’s Dis..

[78] Schallier A., Smolders I., Van Dam D., Loyens E., De Deyn P.P., Michotte A., Michotte Y., Massie A. (2011). Region- and age-specific changes in glutamate transport in the AbetaPP23 mouse model for Alzheimer’s disease. J. Alzheimer’s Dis..

[79] Scimemi A., Meabon J.S., Woltjer R.L., Sullivan J.M., Diamond J.S., Cook D.G. (2013). Amyloid-beta1-42 slows clearance of synaptically released glutamate by mislocalizing astrocytic GLT-1. J. Neurosci. Off. J. Soc. Neurosci..

[80] Lauderback C.M., Hackett J.M., Huang F.F., Keller J.N., Szweda L.I., Markesbery W.R., Butterfield D.A. (2001). The glial glutamate transporter, GLT-1, is oxidatively modified by 4-hydroxy-2-nonenal in the Alzheimer’s disease brain: The role of Abeta1-42. J. Neurochem..

[81] Romano A., Serviddio G., Calcagnini S., Villani R., Giudetti A.M., Cassano T., Gaetani S. (2017). Linking lipid peroxidation and neuropsychiatric disorders: Focus on 4-hydroxy-2-nonenal. Free Radic. Biol. Med..

[82] Di Domenico F., Tramutola A., Butterfield D.A. (2017). Role of 4-hydroxy-2-nonenal (HNE) in the pathogenesis of alzheimer disease and other selected age-related neurodegenerative disorders. Free Radic. Biol. Med..

[83] Meeker K.D., Meabon J.S., Cook D.G. (2015). Partial Loss of the Glutamate Transporter GLT-1 Alters Brain Akt and Insulin Signaling in a Mouse Model of Alzheimer’s Disease. J. Alzheimer’s Dis..

[84] Traynelis S.F., Wollmuth L.P., McBain C.J., Menniti F.S., Vance K.M., Ogden K.K., Hansen K.B., Yuan H., Myers S.J., Dingledine R. (2010). Glutamate receptor ion channels: Structure, regulation, and function. Pharmacol. Rev..

[85] Calabresi P., Pisani A., Mercuri N.B., Bernardi G. (1992). Long-term Potentiation in the Striatum is Unmasked by Removing the Voltage-dependent Magnesium Block of NMDA Receptor Channels. Eur. J. Neurosci..

[86] Pandis C., Sotiriou E., Kouvaras E., Asprodini E., Papatheodoropoulos C., Angelatou F. (2006). Differential expression of NMDA and AMPA receptor subunits in rat dorsal and ventral hippocampus. Neuroscience.

[87] Wisden W., Seeburg P.H. (1993). Mammalian ionotropic glutamate receptors. Curr. Opin. Neurobiol..

[88] Brickley S.G., Misra C., Mok M.H., Mishina M., Cull-Candy S.G. (2003). NR2B and NR2D subunits coassemble in cerebellar Golgi cells to form a distinct NMDA receptor subtype restricted to extrasynaptic sites. J. Neurosci..

[89] Paoletti P., Bellone C., Zhou Q. (2013). NMDA receptor subunit diversity: Impact on receptor properties, synaptic plasticity and disease. Nat. Rev. Neurosci..

[90] Rudy C.C., Hunsberger H.C., Weitzner D.S., Reed M.N. (2015). The role of the tripartite glutamatergic synapse in the pathophysiology of Alzheimer’s disease. Aging Dis..

[91] Chater T.E., Goda Y. (2014). The role of AMPA receptors in postsynaptic mechanisms of synaptic plasticity. Front. Cell Neurosci..

[92] Fabian-Fine R., Volknandt W., Fine A., Stewart M.G. (2000). Age-dependent pre- and postsynaptic distribution of AMPA receptors at synapses in CA3 stratum radiatum of hippocampal slice cultures compared with intact brain. Eur. J. Neurosci..

[93] Schenk U., Matteoli M. (2004). Presynaptic AMPA receptors: More than just ion channels?. Biol. Cell.

[94] Schoepp D.D. (2001). Unveiling the functions of presynaptic metabotropic glutamate receptors in the central nervous system. J. Pharmacol. Exp. Ther..

[95] Lea P.M., Custer S.J., Vicini S., Faden A.I. (2002). Neuronal and glial mGluR5 modulation prevents stretch-induced enhancement of NMDA receptor current. Pharmacol. Biochem. Behav..

[96] Skeberdis V.A., Lan J., Opitz T., Zheng X., Bennett M.V., Zukin R.S. (2001). mGluR1-mediated potentiation of NMDA receptors involves a rise in intracellular calcium and activation of protein kinase C. Neuropharmacology.

[97] Rubio M.D., Drummond J.B., Meador-Woodruff J.H. (2012). Glutamate receptor abnormalities in schizophrenia: Implications for innovative treatments. Biomol. Ther..

[98] Ferraguti F., Shigemoto R. (2006). Metabotropic glutamate receptors. Cell Tissue Res..

[99] Holmes A., Spanagel R., Krystal J.H. (2013). Glutamatergic targets for new alcohol medications. Psychopharmacology.

[100] Lutgen V., Qualmann K., Resch J., Kong L., Choi S., Baker D.A. (2013). Reduction in phencyclidine induced sensorimotor gating deficits in the rat following increased system xc(-) activity in the medial prefrontal cortex. Psychopharmacology.

[101] Ambrosini A., Bresciani L., Fracchia S., Brunello N., Racagni G. (1995). Metabotropic glutamate receptors negatively coupled to adenylate cyclase inhibit N-methyl-D-aspartate receptor activity and prevent neurotoxicity in mesencephalic neurons in vitro. Mol. Pharmacol..

[102] Pinheiro P.S., Mulle C. (2008). Presynaptic glutamate receptors: Physiological functions and mechanisms of action. Nat. Rev. Neurosci..

[103] Paoletti P. (2011). Molecular basis of NMDA receptor functional diversity. Eur. J. Neurosci..

[104] Kohr G. (2006). NMDA receptor function: Subunit composition versus spatial distribution. Cell Tissue Res..

[105] Hardingham G.E., Fukunaga Y., Bading H. (2002). Extrasynaptic NMDARs oppose synaptic NMDARs by triggering CREB shut-off and cell death pathways. Nat. Neurosci..

[106] Wang Z.C., Zhao J., Li S. (2013). Dysregulation of synaptic and extrasynaptic N-methyl-D-aspartate receptors induced by amyloid-beta. Neurosci. Bull..

[107] Bordji K., Becerril-Ortega J., Nicole O., Buisson A. (2010). Activation of extrasynaptic, but not synaptic, NMDA receptors modifies amyloid precursor protein expression pattern and increases amyloid-ss production. J. Neurosci. Off. J. Soc. Neurosci..

[108] Mayer M.L., Westbrook G.L., Guthrie P.B. (1984). Voltage-dependent block by Mg2+ of NMDA responses in spinal cord neurones. Nature.

[109] Esposito Z., Belli L., Toniolo S., Sancesario G., Bianconi C., Martorana A. (2013). Amyloid beta, glutamate, excitotoxicity in Alzheimer’s disease: Are we on the right track?. CNS Neurosci. Ther..

[110] Kullmann D.M., Lamsa K.P. (2007). Long-term synaptic plasticity in hippocampal interneurons. Nat. Rev. Neurosci..

[111] Siman R., Noszek J.C. (1988). Excitatory amino acids activate calpain I and induce structural protein breakdown in vivo. Neuron.

[112] Lazarewicz J.W., Wroblewski J.T., Costa E. (1990). N-methyl-D-aspartate-sensitive glutamate receptors induce calcium-mediated arachidonic acid release in primary cultures of cerebellar granule cells. J. Neurochem..

[113] Chan P.H., Fishman R.A. (1978). Brain edema: Induction in cortical slices by polyunsaturated fatty acids. Science.

[114] Di Meo S., Reed T.T., Venditti P., Victor V.M. (2016). Role of ROS and RNS Sources in Physiological and Pathological Conditions. Oxidative Med. Cell. Longev..

[115] Gong C.X., Liu F., Grundke-Iqbal I., Iqbal K. (2005). Post-translational modifications of tau protein in Alzheimer’s disease. J. Neural Transm..

[116] Mattson M.P. (1992). Effects of microtubule stabilization and destabilization on tau immunoreactivity in cultured hippocampal neurons. Brain Res..

[117] Wang R., Reddy P.H. (2017). Role of Glutamate and NMDA Receptors in Alzheimer’s Disease. J. Alzheimer’s Dis..

[118] Glabe C.G. (2008). Structural classification of toxic amyloid oligomers. J. Biol. Chem..

[119] Shankar G.M., Bloodgood B.L., Townsend M., Walsh D.M., Selkoe D.J., Sabatini B.L. (2007). Natural oligomers of the Alzheimer amyloid-beta protein induce reversible synapse loss by modulating an NMDA-type glutamate receptor-dependent signaling pathway. J. Neurosci. Off. J. Soc. Neurosci..

[120] Wu H.Y., Hudry E., Hashimoto T., Kuchibhotla K., Rozkalne A., Fan Z., Spires-Jones T., Xie H., Arbel-Ornath M., Grosskreutz C.L. (2010). Amyloid beta induces the morphological neurodegenerative triad of spine loss, dendritic simplification, and neuritic dystrophies through calcineurin activation. J. Neurosci. Off. J. Soc. Neurosci..

[121] Takai H., Katayama K., Uetsuka K., Nakayama H., Doi K. (2003). Distribution of N-methyl-D-aspartate receptors (NMDARs) in the developing rat brain. Exp. Mol. Pathol..

[122] Malinow R. (2012). New developments on the role of NMDA receptors in Alzheimer’s disease. Curr. Opin. Neurobiol..

[123] Brito-Moreira J., Paula-Lima A.C., Bomfim T.R., Oliveira F.B., Sepulveda F.J., De Mello F.G., Aguayo L.G., Panizzutti R., Ferreira S.T. (2011). Abeta oligomers induce glutamate release from hippocampal neurons. Curr. Alzheimer Res..

[124] Talantova M., Sanz-Blasco S., Zhang X., Xia P., Akhtar M.W., Okamoto S., Dziewczapolski G., Nakamura T., Cao G., Pratt A.E. (2013). Aβ induces astrocytic glutamate release, extrasynaptic NMDA receptor activation, and synaptic loss. Proc. Natl. Acad. Sci. USA.

[125] Hsieh H., Boehm J., Sato C., Iwatsubo T., Tomita T., Sisodia S., Malinow R. (2006). AMPAR removal underlies Abeta-induced synaptic depression and dendritic spine loss. Neuron.

[126] Li S., Jin M., Koeglsperger T., Shepardson N.E., Shankar G.M., Selkoe D.J. (2011). Soluble Abeta oligomers inhibit long-term potentiation through a mechanism involving excessive activation of extrasynaptic NR2B-containing NMDA receptors. J. Neurosci. Off. J. Soc. Neurosci..

[127] Wang Q., Walsh D.M., Rowan M.J., Selkoe D.J., Anwyl R. (2004). Block of long-term potentiation by naturally secreted and synthetic amyloid beta-peptide in hippocampal slices is mediated via activation of the kinases c-Jun N-terminal kinase, cyclin-dependent kinase 5, and p38 mitogen-activated protein kinase as well as metabotropic glutamate receptor type 5. J. Neurosci. Off. J. Soc. Neurosci..

[128] Calvo-Rodriguez M., Hou S.S., Snyder A.C., Kharitonova E.K., Russ A.N., Das S., Fan Z., Muzikansky A., Garcia-Alloza M., Serrano-Pozo A. (2020). Increased mitochondrial calcium levels associated with neuronal death in a mouse model of Alzheimer’s disease. Nat. Commun..

[129] Muller M., Ahumada-Castro U., Sanhueza M., Gonzalez-Billault C., Court F.A., Cardenas C. (2018). Mitochondria and Calcium Regulation as Basis of Neurodegeneration Associated with Aging. Front. Neurosci..

[130] Perez M.J., Ponce D.P., Aranguiz A., Behrens M.I., Quintanilla R.A. (2018). Mitochondrial permeability transition pore contributes to mitochondrial dysfunction in fibroblasts of patients with sporadic Alzheimer’s disease. Redox Biol..

[131] Akama K.T., Van Eldik L.J. (2000). Beta-amyloid stimulation of inducible nitric-oxide synthase in astrocytes is interleukin-1beta- and tumor necrosis factor-alpha (TNFalpha)-dependent, and involves a TNFalpha receptor-associated factor- and NFkappaB-inducing kinase-dependent signaling mechanism. J. Biol. Chem..

[132] Scuderi C., Bronzuoli M.R., Facchinetti R., Pace L., Ferraro L., Broad K.D., Serviddio G., Bellanti F., Palombelli G., Carpinelli G. (2018). Ultramicronized palmitoylethanolamide rescues learning and memory impairments in a triple transgenic mouse model of Alzheimer’s disease by exerting anti-inflammatory and neuroprotective effects. Transl. Psychiatry.

[133] Wang W.Y., Tan M.S., Yu J.T., Tan L. (2015). Role of pro-inflammatory cytokines released from microglia in Alzheimer’s disease. Ann. Transl. Med..

[134] Verges D.K., Restivo J.L., Goebel W.D., Holtzman D.M., Cirrito J.R. (2011). Opposing synaptic regulation of amyloid-beta metabolism by NMDA receptors in vivo. J. Neurosci. Off. J. Soc. Neurosci..

[135] Shi X.D., Sun K., Hu R., Liu X.Y., Hu Q.M., Sun X.Y., Yao B., Sun N., Hao J.R., Wei P. (2016). Blocking the Interaction between EphB2 and ADDLs by a Small Peptide Rescues Impaired Synaptic Plasticity and Memory Deficits in a Mouse Model of Alzheimer’s Disease. J. Neurosci. Off. J. Soc. Neurosci..

[136] Kurup P., Zhang Y., Xu J., Venkitaramani D.V., Haroutunian V., Greengard P., Nairn A.C., Lombroso P.J. (2010). Abeta-mediated NMDA receptor endocytosis in Alzheimer’s disease involves ubiquitination of the tyrosine phosphatase STEP61. J. Neurosci. Off. J. Soc. Neurosci..

[137] Snyder E.M., Nong Y., Almeida C.G., Paul S., Moran T., Choi E.Y., Nairn A.C., Salter M.W., Lombroso P.J., Gouras G.K. (2005). Regulation of NMDA receptor trafficking by amyloid-beta. Nat. Neurosci..

[138] Zhang Y., Kurup P., Xu J., Anderson G.M., Greengard P., Nairn A.C., Lombroso P.J. (2011). Reduced levels of the tyrosine phosphatase STEP block beta amyloid-mediated GluA1/GluA2 receptor internalization. J. Neurochem..

[139] Lauren J., Gimbel D.A., Nygaard H.B., Gilbert J.W., Strittmatter S.M. (2009). Cellular prion protein mediates impairment of synaptic plasticity by amyloid-beta oligomers. Nature.

[140] You H., Tsutsui S., Hameed S., Kannanayakal T.J., Chen L., Xia P., Engbers J.D., Lipton S.A., Stys P.K., Zamponi G.W. (2012). Abeta neurotoxicity depends on interactions between copper ions, prion protein, and N-methyl-D-aspartate receptors. Proc. Natl. Acad. Sci. USA.

[141] Hoover B.R., Reed M.N., Su J., Penrod R.D., Kotilinek L.A., Grant M.K., Pitstick R., Carlson G.A., Lanier L.M., Yuan L.L. (2010). Tau mislocalization to dendritic spines mediates synaptic dysfunction independently of neurodegeneration. Neuron.

[142] Mondragón-Rodríguez S., Trillaud-Doppia E., Dudilot A., Bourgeois C., Lauzon M., Leclerc N., Boehm J. (2012). Interaction of endogenous tau protein with synaptic proteins is regulated by N-methyl-D-aspartate receptor-dependent tau phosphorylation. J. Biol. Chem..

[143] Nakazawa T., Komai S., Tezuka T., Hisatsune C., Umemori H., Semba K., Mishina M., Manabe T., Yamamoto T. (2001). Characterization of Fyn-mediated tyrosine phosphorylation sites on GluR epsilon 2 (NR2B) subunit of the N-methyl-D-aspartate receptor. J. Biol. Chem..

[144] Roberson E.D., Scearce-Levie K., Palop J.J., Yan F., Cheng I.H., Wu T., Gerstein H., Yu G.Q., Mucke L. (2007). Reducing endogenous tau ameliorates amyloid beta-induced deficits in an Alzheimer’s disease mouse model. Science.

[145] De Montigny A., Elhiri I., Allyson J., Cyr M., Massicotte G. (2013). NMDA reduces Tau phosphorylation in rat hippocampal slices by targeting NR2A receptors, GSK3beta, and PKC activities. Neural Plast..

[146] Roberson E.D., Halabisky B., Yoo J.W., Yao J., Chin J., Yan F., Wu T., Hamto P., Devidze N., Yu G.Q. (2011). Amyloid-beta/Fyn-induced synaptic, network, and cognitive impairments depend on tau levels in multiple mouse models of Alzheimer’s disease. J. Neurosci. Off. J. Soc. Neurosci..

[147] Lu W., Shi Y., Jackson A.C., Bjorgan K., During M.J., Sprengel R., Seeburg P.H., Nicoll R.A. (2009). Subunit composition of synaptic AMPA receptors revealed by a single-cell genetic approach. Neuron.

[148] Anggono V., Huganir R.L. (2012). Regulation of AMPA receptor trafficking and synaptic plasticity. Curr. Opin. Neurobiol..

[149] Seo J., Giusti-Rodriguez P., Zhou Y., Rudenko A., Cho S., Ota K.T., Park C., Patzke H., Madabhushi R., Pan L. (2014). Activity-dependent p25 generation regulates synaptic plasticity and Abeta-induced cognitive impairment. Cell.

[150] Bibb J.A., Snyder G.L., Nishi A., Yan Z., Meijer L., Fienberg A.A., Tsai L.H., Kwon Y.T., Girault J.A., Czernik A.J. (1999). Phosphorylation of DARPP-32 by Cdk5 modulates dopamine signalling in neurons. Nature.

[151] Guntupalli S., Widagdo J., Anggono V. (2016). Amyloid-beta-Induced Dysregulation of AMPA Receptor Trafficking. Neural Plast..

[152] Cruz J.C., Kim D., Moy L.Y., Dobbin M.M., Sun X., Bronson R.T., Tsai L.H. (2006). p25/cyclin-dependent kinase 5 induces production and intraneuronal accumulation of amyloid beta in vivo. J. Neurosci. Off. J. Soc. Neurosci..

[153] Fu A.K., Fu W.Y., Ng A.K., Chien W.W., Ng Y.P., Wang J.H., Ip N.Y. (2004). Cyclin-dependent kinase 5 phosphorylates signal transducer and activator of transcription 3 and regulates its transcriptional activity. Proc. Natl. Acad. Sci. USA.

[154] Wen Y., Yu W.H., Maloney B., Bailey J., Ma J., Marie I., Maurin T., Wang L., Figueroa H., Herman M. (2008). Transcriptional regulation of beta-secretase by p25/cdk5 leads to enhanced amyloidogenic processing. Neuron.

[155] Sun K.H., de Pablo Y., Vincent F., Johnson E.O., Chavers A.K., Shah K. (2008). Novel genetic tools reveal Cdk5’s major role in Golgi fragmentation in Alzheimer’s disease. Mol. Biol. Cell.

[156] Zempel H., Thies E., Mandelkow E., Mandelkow E.M. (2010). Abeta oligomers cause localized Ca(2+) elevation, missorting of endogenous Tau into dendrites, Tau phosphorylation, and destruction of microtubules and spines. J. Neurosci. Off. J. Soc. Neurosci..

[157] Zhao D., Watson J.B., Xie C.W. (2004). Amyloid beta prevents activation of calcium/calmodulin-dependent protein kinase II and AMPA receptor phosphorylation during hippocampal long-term potentiation. J. Neurophysiol..

[158] Kristensen A.S., Jenkins M.A., Banke T.G., Schousboe A., Makino Y., Johnson R.C., Huganir R., Traynelis S.F. (2011). Mechanism of Ca2+/calmodulin-dependent kinase II regulation of AMPA receptor gating. Nat. Neurosci..

[159] Tomita S., Stein V., Stocker T.J., Nicoll R.A., Bredt D.S. (2005). Bidirectional synaptic plasticity regulated by phosphorylation of stargazin-like TARPs. Neuron.

[160] Zhu J.J., Qin Y., Zhao M., Van Aelst L., Malinow R. (2002). Ras and Rap control AMPA receptor trafficking during synaptic plasticity. Cell.

[161] Gu Z., Liu W., Yan Z. (2009). {beta}-Amyloid impairs AMPA receptor trafficking and function by reducing Ca2+/calmodulin-dependent protein kinase II synaptic distribution. J. Biol. Chem..

[162] Pin J.P., Galvez T., Prezeau L. (2003). Evolution, structure, and activation mechanism of family 3/C G-protein-coupled receptors. Pharmacol. Ther..

[163] Chuang S.C., Bianchi R., Wong R.K. (2000). Group I mGluR activation turns on a voltage-gated inward current in hippocampal pyramidal cells. J. Neurophysiol..

[164] Ferraguti F., Baldani-Guerra B., Corsi M., Nakanishi S., Corti C. (1999). Activation of the extracellular signal-regulated kinase 2 by metabotropic glutamate receptors. Eur. J. Neurosci..

[165] Iacovelli L., Bruno V., Salvatore L., Melchiorri D., Gradini R., Caricasole A., Barletta E., De Blasi A., Nicoletti F. (2002). Native group-III metabotropic glutamate receptors are coupled to the mitogen-activated protein kinase/phosphatidylinositol-3-kinase pathways. J. Neurochem..

[166] D’Antoni S., Berretta A., Bonaccorso C.M., Bruno V., Aronica E., Nicoletti F., Catania M.V. (2008). Metabotropic glutamate receptors in glial cells. Neurochem. Res..

[167] Ribeiro F.M., Vieira L.B., Pires R.G., Olmo R.P., Ferguson S.S. (2017). Metabotropic glutamate receptors and neurodegenerative diseases. Pharmacol. Res..

[168] Song M.S., Rauw G., Baker G.B., Kar S. (2008). Memantine protects rat cortical cultured neurons against beta-amyloid-induced toxicity by attenuating tau phosphorylation. Eur. J. Neurosci..

[169] D’Amelio M., Cavallucci V., Middei S., Marchetti C., Pacioni S., Ferri A., Diamantini A., De Zio D., Carrara P., Battistini L. (2011). Caspase-3 triggers early synaptic dysfunction in a mouse model of Alzheimer’s disease. Nat. Neurosci..

[170] Brody A.H., Strittmatter S.M. (2018). Synaptotoxic Signaling by Amyloid Beta Oligomers in Alzheimer’s Disease Through Prion Protein and mGluR5. Adv. Pharm..

[171] Bruno V., Caraci F., Copani A., Matrisciano F., Nicoletti F., Battaglia G. (2017). The impact of metabotropic glutamate receptors into active neurodegenerative processes: A “dark side” in the development of new symptomatic treatments for neurologic and psychiatric disorders. Neuropharmacology.

[172] Won H., Lee H.R., Gee H.Y., Mah W., Kim J.I., Lee J., Ha S., Chung C., Jung E.S., Cho Y.S. (2012). Autistic-like social behaviour in Shank2-mutant mice improved by restoring NMDA receptor function. Nature.

[173] Renner M., Lacor P.N., Velasco P.T., Xu J., Contractor A., Klein W.L., Triller A. (2010). Deleterious effects of amyloid beta oligomers acting as an extracellular scaffold for mGluR5. Neuron.

[174] Zhang H., Wu L., Pchitskaya E., Zakharova O., Saito T., Saido T., Bezprozvanny I. (2015). Neuronal Store-Operated Calcium Entry and Mushroom Spine Loss in Amyloid Precursor Protein Knock-In Mouse Model of Alzheimer’s Disease. J. Neurosci. Off. J. Soc. Neurosci..

[175] Matta J.A., Ashby M.C., Sanz-Clemente A., Roche K.W., Isaac J.T. (2011). mGluR5 and NMDA receptors drive the experience- and activity-dependent NMDA receptor NR2B to NR2A subunit switch. Neuron.

[176] Tu J.C., Xiao B., Naisbitt S., Yuan J.P., Petralia R.S., Brakeman P., Doan A., Aakalu V.K., Lanahan A.A., Sheng M. (1999). Coupling of mGluR/Homer and PSD-95 complexes by the Shank family of postsynaptic density proteins. Neuron.

[177] Lujan R., Nusser Z., Roberts J.D., Shigemoto R., Somogyi P. (1996). Perisynaptic location of metabotropic glutamate receptors mGluR1 and mGluR5 on dendrites and dendritic spines in the rat hippocampus. Eur. J. Neurosci..

[178] Shigemoto R., Kinoshita A., Wada E., Nomura S., Ohishi H., Takada M., Flor P.J., Neki A., Abe T., Nakanishi S. (1997). Differential presynaptic localization of metabotropic glutamate receptor subtypes in the rat hippocampus. J. Neurosci. Off. J. Soc. Neurosci..

[179] Shigemoto R., Nomura S., Ohishi H., Sugihara H., Nakanishi S., Mizuno N. (1993). Immunohistochemical localization of a metabotropic glutamate receptor, mGluR5, in the rat brain. Neurosci. Lett..

[180] Bruno V., Ksiazek I., Battaglia G., Lukic S., Leonhardt T., Sauer D., Gasparini F., Kuhn R., Nicoletti F., Flor P.J. (2000). Selective blockade of metabotropic glutamate receptor subtype 5 is neuroprotective. Neuropharmacology.

[181] Hamilton A., Esseltine J.L., DeVries R.A., Cregan S.P., Ferguson S.S. (2014). Metabotropic glutamate receptor 5 knockout reduces cognitive impairment and pathogenesis in a mouse model of Alzheimer’s disease. Mol. Brain.

[182] Kingston A.E., O’Neill M.J., Bond A., Bruno V., Battaglia G., Nicoletti F., Harris J.R., Clark B.P., Monn J.A., Lodge D. (1999). Neuroprotective actions of novel and potent ligands of group I and group II metabotropic glutamate receptors. Ann. N. Y. Acad. Sci..

[183] Movsesyan V.A., O’Leary D.M., Fan L., Bao W., Mullins P.G., Knoblach S.M., Faden A.I. (2001). mGluR5 antagonists 2-methyl-6-(phenylethynyl)-pyridine and (E)-2-methyl-6-(2-phenylethenyl)-pyridine reduce traumatic neuronal injury in vitro and in vivo by antagonizing N-methyl-D-aspartate receptors. J. Pharmacol. Exp. Ther..

[184] Overk C.R., Cartier A., Shaked G., Rockenstein E., Ubhi K., Spencer B., Price D.L., Patrick C., Desplats P., Masliah E. (2014). Hippocampal neuronal cells that accumulate alpha-synuclein fragments are more vulnerable to Abeta oligomer toxicity via mGluR5--implications for dementia with Lewy bodies. Mol. Neurodegener..

[185] Selkoe D.J. (2000). Toward a comprehensive theory for Alzheimer’s disease. Hypothesis: Alzheimer’s disease is caused by the cerebral accumulation and cytotoxicity of amyloid beta-protein. Ann. N. Y. Acad. Sci..

[186] Haas L.T., Kostylev M.A., Strittmatter S.M. (2014). Therapeutic molecules and endogenous ligands regulate the interaction between brain cellular prion protein (PrPC) and metabotropic glutamate receptor 5 (mGluR5). J. Biol. Chem..

[187] Um J.W., Kaufman A.C., Kostylev M., Heiss J.K., Stagi M., Takahashi H., Kerrisk M.E., Vortmeyer A., Wisniewski T., Koleske A.J. (2013). Metabotropic glutamate receptor 5 is a coreceptor for Alzheimer abeta oligomer bound to cellular prion protein. Neuron.

[188] Hamilton A., Zamponi G.W., Ferguson S.S. (2015). Glutamate receptors function as scaffolds for the regulation of beta-amyloid and cellular prion protein signaling complexes. Mol. Brain.

[189] Dohler F., Sepulveda-Falla D., Krasemann S., Altmeppen H., Schluter H., Hildebrand D., Zerr I., Matschke J., Glatzel M. (2014). High molecular mass assemblies of amyloid-beta oligomers bind prion protein in patients with Alzheimer’s disease. Brain.

[190] Kessels H.W., Nguyen L.N., Nabavi S., Malinow R. (2010). The prion protein as a receptor for amyloid-beta. Nature.

[191] Riedel G., Platt B., Micheau J. (2003). Glutamate receptor function in learning and memory. Behav. Brain res..

[192] Berent-Spillson A., Russell J.W. (2007). Metabotropic glutamate receptor 3 protects neurons from glucose-induced oxidative injury by increasing intracellular glutathione concentration. J. Neurochem..

[193] Caraci F., Molinaro G., Battaglia G., Giuffrida M.L., Riozzi B., Traficante A., Bruno V., Cannella M., Merlo S., Wang X. (2011). Targeting group II metabotropic glutamate (mGlu) receptors for the treatment of psychosis associated with Alzheimer’s disease: Selective activation of mGlu2 receptors amplifies beta-amyloid toxicity in cultured neurons, whereas dual activation of mGlu2 and mGlu3 receptors is neuroprotective. Mol. Pharmacol..

[194] Pinteaux-Jones F., Sevastou I.G., Fry V.A., Heales S., Baker D., Pocock J.M. (2008). Myelin-induced microglial neurotoxicity can be controlled by microglial metabotropic glutamate receptors. J. Neurochem..

[195] Durand D., Carniglia L., Beauquis J., Caruso C., Saravia F., Lasaga M. (2014). Astroglial mGlu3 receptors promote alpha-secretase-mediated amyloid precursor protein cleavage. Neuropharmacology.

[196] Taylor D.L., Jones F., Kubota E.S., Pocock J.M. (2005). Stimulation of microglial metabotropic glutamate receptor mGlu2 triggers tumor necrosis factor alpha-induced neurotoxicity in concert with microglial-derived Fas ligand. J. Neurosci. Off. J. Soc. Neurosci..

[197] Durand D., Carniglia L., Turati J., Ramirez D., Saba J., Caruso C., Lasaga M. (2017). Amyloid-beta neurotoxicity and clearance are both regulated by glial group II metabotropic glutamate receptors. Neuropharmacology.

[198] Goeldner C., Ballard T.M., Knoflach F., Wichmann J., Gatti S., Umbricht D. (2013). Cognitive impairment in major depression and the mGlu2 receptor as a therapeutic target. Neuropharmacology.

[199] Higgins G.A., Ballard T.M., Kew J.N., Richards J.G., Kemp J.A., Adam G., Woltering T., Nakanishi S., Mutel V. (2004). Pharmacological manipulation of mGlu2 receptors influences cognitive performance in the rodent. Neuropharmacology.

[200] Taylor D.L., Diemel L.T., Cuzner M.L., Pocock J.M. (2002). Activation of group II metabotropic glutamate receptors underlies microglial reactivity and neurotoxicity following stimulation with chromogranin A, a peptide up-regulated in Alzheimer’s disease. J. Neurochem..

[201] Caraci F., Gulisano W., Guida C.A., Impellizzeri A.A., Drago F., Puzzo D., Palmeri A. (2015). A key role for TGF-beta1 in hippocampal synaptic plasticity and memory. Sci. Rep..

[202] Tichauer J.E., von Bernhardi R. (2012). Transforming growth factor-beta stimulates beta amyloid uptake by microglia through Smad3-dependent mechanisms. J. Neurosci. Res..

[203] Das P., Golde T. (2006). Dysfunction of TGF-beta signaling in Alzheimer’s disease. J. Clin. Investig..

[204] Tesseur I., Zou K., Esposito L., Bard F., Berber E., Can J.V., Lin A.H., Crews L., Tremblay P., Mathews P. (2006). Deficiency in neuronal TGF-beta signaling promotes neurodegeneration and Alzheimer’s pathology. J. Clin. Investig..

[205] Neale J.H., Olszewski R.T., Gehl L.M., Wroblewska B., Bzdega T. (2005). The neurotransmitter N-acetylaspartylglutamate in models of pain, ALS, diabetic neuropathy, CNS injury and schizophrenia. Trends Pharmacol. Sci..

[206] Neale J.H., Olszewski R.T., Zuo D., Janczura K.J., Profaci C.P., Lavin K.M., Madore J.C., Bzdega T. (2011). Advances in understanding the peptide neurotransmitter NAAG and appearance of a new member of the NAAG neuropeptide family. J. Neurochem..

[207] Janczura K.J., Olszewski R.T., Bzdega T., Bacich D.J., Heston W.D., Neale J.H. (2013). NAAG peptidase inhibitors and deletion of NAAG peptidase gene enhance memory in novel object recognition test. Eur. J. Pharmacol..

[208] Olszewski R.T., Janczura K.J., Bzdega T., Der E.K., Venzor F., O’Rourke B., Hark T.J., Craddock K.E., Balasubramanian S., Moussa C. (2017). NAAG Peptidase Inhibitors Act via mGluR3: Animal Models of Memory, Alzheimer’s, and Ethanol Intoxication. Neurochem. Res..

[209] Cummings J., Lee G., Ritter A., Sabbagh M., Zhong K. (2019). Alzheimer’s disease drug development pipeline: 2019. Alzheimer’s Dementia.

[210] Knight R., Khondoker M., Magill N., Stewart R., Landau S. (2018). A Systematic Review and Meta-Analysis of the Effectiveness of Acetylcholinesterase Inhibitors and Memantine in Treating the Cognitive Symptoms of Dementia. Dementia Geriatr. Cogn. Disord..

[211] Li L., Sengupta A., Haque N., Grundke-Iqbal I., Iqbal K. (2004). Memantine inhibits and reverses the Alzheimer type abnormal hyperphosphorylation of tau and associated neurodegeneration. FEBS Lett..

[212] Möbius H.J., Stöffler A., Graham S.M. (2004). Memantine hydrochloride: Pharmacological and clinical profile. Drugs Today.

[213] van Marum R.J. (2009). Update on the use of memantine in Alzheimer’s disease. Neuropsychiatr. Dis Treat..

[214] Ellison G. (1995). The N-methyl-D-aspartate antagonists phencyclidine, ketamine and dizocilpine as both behavioral and anatomical models of the dementias. Brain Res. Rev..

[215] Xu R., Wang J., Xu J., Song X., Huang H. (2020). Rhynchophylline Loaded-mPEG-PLGA Nanoparticles Coated with Tween-80 for Preliminary Study in Alzheimer’s Disease. Int. J. Nanomed..

[216] Yang Y., Ji W.G., Zhu Z.R., Wu Y.L., Zhang Z.Y., Qu S.C. (2018). Rhynchophylline suppresses soluble Abeta1-42-induced impairment of spatial cognition function via inhibiting excessive activation of extrasynaptic NR2B-containing NMDA receptors. Neuropharmacology.

[217] Ip F.C., Fu W.Y., Cheng E.Y., Tong E.P., Lok K.C., Liang Y., Ye W.C., Ip N.Y. (2015). Anemoside A3 Enhances Cognition through the Regulation of Synaptic Function and Neuroprotection. Neuropsychopharmacology.

[218] Hamilton A., Vasefi M., Vander Tuin C., McQuaid R.J., Anisman H., Ferguson S.S. (2016). Chronic Pharmacological mGluR5 Inhibition Prevents Cognitive Impairment and Reduces Pathogenesis in an Alzheimer Disease Mouse Model. Cell Rep..

[219] Haas L.T., Salazar S.V., Smith L.M., Zhao H.R., Cox T.O., Herber C.S., Degnan A.P., Balakrishnan A., Macor J.E., Albright C.F. (2017). Silent Allosteric Modulation of mGluR5 Maintains Glutamate Signaling while Rescuing Alzheimer’s Mouse Phenotypes. Cell Rep..

[220] Chen X., Lin R., Chang L., Xu S., Wei X., Zhang J., Wang C., Anwyl R., Wang Q. (2013). Enhancement of long-term depression by soluble amyloid beta protein in rat hippocampus is mediated by metabotropic glutamate receptor and involves activation of p38MAPK, STEP and caspase-3. Neuroscience.

[221] Battaglia G., Molinaro G., Riozzi B., Storto M., Busceti C.L., Spinsanti P., Bucci D., Di Liberto V., Mudo G., Corti C. (2009). Activation of mGlu3 receptors stimulates the production of GDNF in striatal neurons. PLoS ONE.

[222] Battaglia G., Riozzi B., Bucci D., Di Menna L., Molinaro G., Pallottino S., Nicoletti F., Bruno V. (2015). Activation of mGlu3 metabotropic glutamate receptors enhances GDNF and GLT-1 formation in the spinal cord and rescues motor neurons in the SOD-1 mouse model of amyotrophic lateral sclerosis. Neurobiol. Dis..

[223] Corti C., Battaglia G., Molinaro G., Riozzi B., Pittaluga A., Corsi M., Mugnaini M., Nicoletti F., Bruno V. (2007). The use of knock-out mice unravels distinct roles for mGlu2 and mGlu3 metabotropic glutamate receptors in mechanisms of neurodegeneration/neuroprotection. J. Neurosci. Off. J. Soc. Neurosci..

[224] Kim S.H., Fraser P.E., Westaway D., St George-Hyslop P.H., Ehrlich M.E., Gandy S. (2010). Group II metabotropic glutamate receptor stimulation triggers production and release of Alzheimer’s amyloid(beta)42 from isolated intact nerve terminals. J. Neurosci. Off. J. Soc. Neurosci..

[225] Kortagere S., Mortensen O.V., Xia J., Lester W., Fang Y., Srikanth Y., Salvino J.M., Fontana A.C.K. (2018). Identification of Novel Allosteric Modulators of Glutamate Transporter EAAT2. ACS Chem. Neurosci..

[226] Danysz W., Parsons C.G., Mobius H.J., Stoffler A., Quack G. (2000). Neuroprotective and symptomatological action of memantine relevant for Alzheimer’s disease—A unified glutamatergic hypothesis on the mechanism of action. Neurotox. Res..

[227] Alam S., Lingenfelter K.S., Bender A.M., Lindsley C.W. (2017). Classics in Chemical Neuroscience: Memantine. ACS Chem. Neurosci..

[228] Abd-Elrahman K.S., Hamilton A., Vasefi M., Ferguson S.S.G. (2018). Autophagy is increased following either pharmacological or genetic silencing of mGluR5 signaling in Alzheimer’s disease mouse models. Mol. Brain.

[229] Abd-Elrahman K.S., Hamilton A., Albaker A., Ferguson S.S.G. (2020). mGluR5 Contribution to Neuropathology in Alzheimer Mice Is Disease Stage-Dependent. ACS Pharmacol. Translat. Sci..

[230] Shrivastava A.N., Kowalewski J.M., Renner M., Bousset L., Koulakoff A., Melki R., Giaume C., Triller A. (2013). beta-amyloid and ATP-induced diffusional trapping of astrocyte and neuronal metabotropic glutamate type-5 receptors. Glia.

[231] Pitsikas N., Kaffe E., Markou A. (2012). The metabotropic glutamate 2/3 receptor antagonist LY341495 differentially affects recognition memory in rats. Behav. Brain Res..

[232] Shimazaki T., Kaku A., Chaki S. (2007). Blockade of the metabotropic glutamate 2/3 receptors enhances social memory via the AMPA receptor in rats. Eur. J. Pharmacol..

[233] Di Liberto V., Bonomo A., Frinchi M., Belluardo N., Mudo G. (2010). Group II metabotropic glutamate receptor activation by agonist LY379268 treatment increases the expression of brain derived neurotrophic factor in the mouse brain. Neuroscience.

[234] Venero J.L., Santiago M., Tomás-Camardiel M., Matarredona E.R., Cano J., Machado A. (2002). DCG-IV but not other group-II metabotropic receptor agonists induces microglial BDNF mRNA expression in the rat striatum. Correlation with neuronal injury. Neuroscience.

